# Characterization of Lipids in Saliva, Tears and Minor Salivary Glands of Sjögren’s Syndrome Patients Using an HPLC/MS-Based Approach

**DOI:** 10.3390/ijms22168997

**Published:** 2021-08-20

**Authors:** Fredrik Fineide, Xiangjun Chen, Thomas Bjellaas, Valeria Vitelli, Tor Paaske Utheim, Janicke Liaaen Jensen, Hilde Kanli Galtung

**Affiliations:** 1 Department of Plastic and Reconstructive Surgery, Oslo University Hospital, 1171 Oslo, Norway; fre_fin@hotmail.com (F.F.); uxutto@ous-hf.no (T.P.U.); 2 The Norwegian Dry Eye Clinic, Ole Vigs Gate 32 E, 0366 Oslo, Norway; 3 Department of Oral Surgery and Oral Medicine, Faculty of Dentistry, University of Oslo, 0317 Oslo, Norway; chenxiangjun1101@gmail.com (X.C.); j.c.l.jensen@odont.uio.no (J.L.J.); 4 VITAS Analytical Services, 0349 Oslo, Norway; tbj@vitas.no; 5 Department of Biostatistics, Institute of Basic Medical Sciences, Faculty of Medicine, University of Oslo, 0316 Oslo, Norway; valeria.vitelli@medisin.uio.no; 6 Department of Medical Biochemistry, Oslo University Hospital, 1171 Oslo, Norway; 7 Department of Oral Biology, Faculty of Dentistry, University of Oslo, 0316 Oslo, Norway

**Keywords:** dry eye disease, eye/retina, inflammation, lipidomics, liquid chromatography, mass spectrometry, meibomian gland dysfunction, Sjögren’s syndrome

## Abstract

The diagnostic work-up of primary Sjögren’s syndrome (pSS) includes quantifying saliva and tear production, evaluation of autoantibodies in serum and histopathological analysis of minor salivary glands. Thus, the potential for further utilizing these fluids and tissues in the quest to find better diagnostic and therapeutic tools should be fully explored. Ten samples of saliva and tears from female patients diagnosed with pSS and ten samples of saliva and tears from healthy females were included for lipidomic analysis of tears and whole saliva using high-performance liquid chromatography coupled to time-of-flight mass spectrometry. In addition, lipidomic analysis was performed on minor salivary gland biopsies from three pSS and three non-SS females. We found significant differences in the lipidomic profiles of saliva and tears in pSS patients compared to healthy controls. Moreover, there were differences in individual lipid species in stimulated saliva that were comparable to those of glandular biopsies, representing an intriguing avenue for further research. We believe a comprehensive elucidation of the changes in lipid composition in saliva, tears and minor salivary glands in pSS patients may be the key to detecting pSS-related dry mouth and dry eyes at an early stage. The identified differences may illuminate the path towards future innovative diagnostic methodologies and treatment modalities for alleviating pSS-related sicca symptoms.

## 1. Introduction

Sjögren’s syndrome (SS) is a chronic, progressive, systemic autoinflammatory disease defined by lymphocytic infiltration of exocrine glands, predominantly the salivary and lacrimal glands [1]. The resultant secretory decrease in saliva and tears often leads to symptoms of dryness in the mouth and eyes (xerostomia and xerophthalmia). SS is considered primary (pSS) when it presents independently, or secondary (sSS) should it occur as part of a previously diagnosed rheumatic disease [2]. SS patients may develop caries, oral fungal infections and systemic symptoms such as fatigue and arthralgia. Additionally, they are at increased risk of acquiring non-Hodgkin’s lymphoma [3]. The etiology of SS is an ongoing matter of debate; however, it is currently believed to result from a combination of genetic, epigenetic, environmental and immunologic factors [4]. The prevalence is estimated to range from 0.03% to 2.7% [5]. Primary Sjögren’s syndrome affects approximately 10 females for every male [6].

The relationship between xerostomia, xerophthalmia and pSS is well established and has previously been the subject of evaluation by our research group [7]. We have demonstrated that critical proteins and cytokines in tears and saliva of pSS patients are upregulated [8,9]. Additionally, we have shown that fat tissue is increased, and genes involved in adipose tissue development are upregulated in the salivary glands of pSS patients [10,11]. To the best of our knowledge, no previous studies have compared the oral and ocular lipidomic profiles of this cohort.

Lipidomics refers to the large-scale analysis of pathways and networks of cellular lipids in biological systems. It is a relatively new field of research that has been driven by rapid advances in technologies. Even with small volumes for analyses, several hundred lipids can be characterized simultaneously. Furthermore, despite the low lipid concentration of fluids such as saliva, novel mass spectrometric devices have reached the level of sensitivity necessary for salivary lipidomics. This rapidly expanding field complements the considerable progress made in genomics and proteomics. Novel lipid biomarkers which may play a role in the pathology of pSS may be identified in saliva and tears by analyzing the compositional changes of lipids.

Saliva is produced by the parotid, submandibular and sublingual glands as well as a plethora of solitary minor glands dispersed within the submucosal layer of the oral cavity [12]. Salivary fluid is composed of water, electrolytes, proteins, lipids, buffers and antimicrobial agents, providing a first line of defense against invading organisms [12,13,14,15]. Additionally, saliva as a whole provides hydration of the teeth and mucous membranes, and facilitates initial food digestion, taste, articulation and swallowing [12]. Lipidomic analysis of saliva has revealed the presence of mono-, di-, and triglycerides, free fatty acids (FFA), squalene, phospholipids, wax esters (WE) and cholesterol esters (CE) [16,17]. Studies indicate that more than half of parotid and submandibular lipid secretions are nonpolar, while the labial salivary glands secrete a greater amount of lipids as well as more polar lipids, such as phospholipids and glycolipids [18,19,20].

Increased oral lipid concentration of general and neutral lipids, specifically FFA and triglycerides, is correlated with susceptibility to caries [17]. However, the functional role of oral lipids has been given little scientific attention and remains poorly understood [21]. Regarding pSS, Slomiany et al. demonstrated a general increase in total salivary lipid count in pSS patients as well as an increased proportion of glycolipids, phospholipids and some neutral lipids [22]. Another study examining eicosanoids reported an increase in prostaglandin E2 and thromboxane B2 in SS patients when compared to healthy controls [23].

The ocular tear film consists of an inner mucoaqueous layer and a superficial lipid layer [24]. Meibum, secreted by the meibomian glands, is the main source of lipids for the lipid layer [24]. The tear film lipid layer can be further divided into an outer nonpolar layer and an inner polar or amphipathic layer [25]. The nonpolar layer consists of mainly WE, CE, triglycerides, diglycerides, monoglycerides and FFA [26,27,28,29]. The composition of the inner amphipathic layer is a matter of ongoing debate. The main polar lipids of the amphiphilic layer seem to be phospholipids and (O-acyl)-ω-hydroxy fatty acids (OAHFA) [30]. However, studies indicate that other sources than meibum, such as the lacrimal gland, lid margin or the lipid bilayer of meibomian gland cells, might be responsible for the presence of phospholipids [31]. The amphipathic layer is hypothesized to generate an interphase between the mucoaqueous layer and the outer nonpolar lipid layer, lowering the surface tension and facilitating spreading of the tear film over the ocular surface with each blink [25,32]. The main role of the nonpolar lipid layer is proposed to be retardation of evaporation [24]. The quality and quantity of lipids in the tear film are essential in maintaining a healthy ocular surface, and deviations in the tear film lipid layer may be both the cause and the result of ocular surface disease and dry eyes. Dysfunction of glands in the eye lids, termed meibomian gland dysfunction (MGD), is one of the most common causes of dry eye disease (DED). Even though MGD is prevalent in SS patients [33], there are few reports on in-depth lipid composition of meibomian glandular fluid by means of lipidomics. Moreover, to the best of our knowledge, no such studies exist concerning pSS patients. This may reflect the combination of high costs and high expertise required to run such analyses.

The current study aims to compare the lipidomic profiles of both saliva and tears between patients suffering from pSS and healthy controls, as well as to evaluate differences in lipidomic profiles of salivary gland biopsies from pSS patients and non-SS sicca controls. Through this, we aim to contribute to the search for potential biomarkers and therapeutic targets in diagnosing and treating patients with the debilitating disease pSS.

## 2. Results

### 2.1. Patient Characteristics

Patients and controls were all aged 30–80 years (mean age patients 57.8 years, mean age controls 52.9 years), had no other diseases that could explain sicca symptomatology and did not use multiple medications influencing saliva and tear production. The ten patients from whom saliva was analyzed had a mean number of 24 teeth (range 16–28) and mean DMFT (decayed, missing due to caries, and filled teeth) of 17 (range 11–27). None had dental implants and only one had a partial denture. Mean unstimulated whole saliva (UWS) flow rates were 0.09 ± 0.05 mL/min and 0.31 ± 0.19 mL/min for pSS and controls, respectively. Mean stimulated whole saliva (SWS) flow rates were 0.68 ± 0.3 mL/min and 1.65 ± 0.71 mL/min for pSS and controls, respectively.

### 2.2. Sphingomyelins and Diacylglycerophosphocholines Are Increased While Diacylglycerols and Ceramides Are Decreased in Unstimulated Whole Saliva from pSS Patients

**Figure 1:**
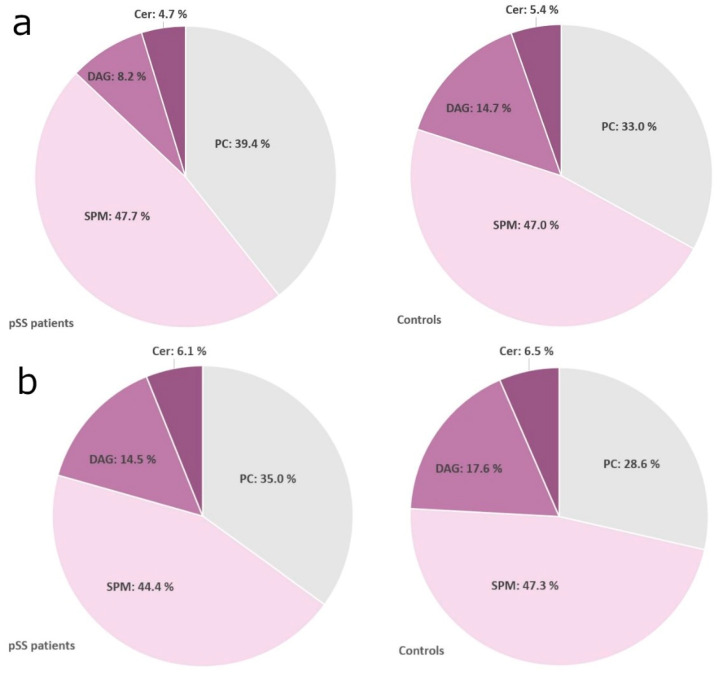
Lipid composition of saliva. (**a**) Unstimulated whole saliva; (**b**) stimulated whole saliva. pSS: primary Sjögren’s syndrome; PC: diacylglycerophosphocholine; SPM: sphingomyelins; DAG: diacylglycerols; Cer: ceramides. The most abundant lipid classes in both unstimulated and stimulated whole saliva were SPM, PC, and DAG, with a smaller contribution of Cer.

In pSS patients, there was a higher average concentration of detected lipids in UWS compared to healthy controls (28.09 µg/mL vs. 8.16 µg/mL, respectively; *p* <0.01). When calculating total lipid output (total lipid concentration × salivary flow rate), the significance between patients and controls disappeared. Furthermore, significant differences between healthy controls and pSS patients were identified in 29/86 individual lipid species in UWS. The lipid composition among pSS patients and healthy controls in UWS is presented in Figure 1. In pSS patients vs. controls, there was an increase in sphingomyelins (SPM) and diacylglycerophosphocholine (PC) as well as a decrease of diacylglycerol (DAG) and ceramides (Cer).

In both groups, 26 individual SPM species were identified, with total fatty acid chain length ranging from C32 to C44. PC were the second most abundant lipid class among both groups, with 21 identified individual lipid species ranging from C30 to C38 in total fatty acid length. Thirdly, 23 DAG species were detected, ranging in total fatty acid length from C32 to C36. Among Cer, 16 species were discovered, with total fatty acid length ranging from C32 to C42. Among all groups, unsaturated fatty acids were in the majority.

**Figure 2:**
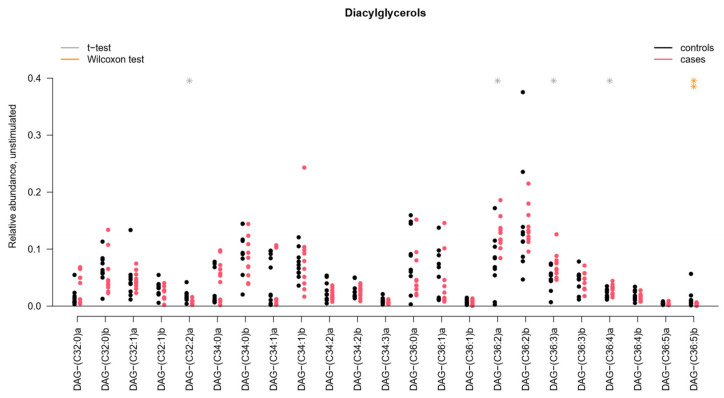
Distribution of individual diacylglycerols (DAG) in unstimulated whole saliva. * *p* < 0.05; ** *p* < 0.01; pSS: primary Sjögren’s syndrome. DAG(C32:2)a, DAG(C36:2)a, DAG(C36:3)a, DAG(C36:4)a, and DAG(C36:5)b showed a significant difference between pSS patients and controls.

Several individual lipids in several lipid classes in UWS were significantly changed: Cer, DAG, PC and SPM. The distribution of individual DAG species detected in UWS is presented in Figure 2; remaining lipid distribution plots can be found in Supplementary Materials as Supplementary Figures S1–S8. Principal component analysis and subsequent multivariate testing showed significant differences between pSS patients and controls in all these aforementioned lipid classes (*p* = 0.02 for Cer, *p* < 0.01 for DAG, *p* < 0.05 for PC and *p* = 0.01 for SPM). No wax esters (WE) or cholesterol esters (CE) were detected in our analysis.

### 2.3. Diacylglycerophosphocholines Are Increased While Sphingomyelins, Diacylglycerols and Ceramides Are Decreased in Stimulated Whole Saliva from pSS Patients

In SWS, the average lipid concentration was 9.66 µg/mL in pSS patients and 3.94 µg/mL in controls (*p* < 0.01). When calculating total lipid output (total lipid concentration × salivary flow rate), the significance between patients and controls disappeared. Significant differences between healthy controls and pSS patients were identified in 28/86 individual lipid species in SWS. The lipid profile of SWS among healthy controls and pSS patients is presented in Figure 1, demonstrating an isolated increase of PC and decrease of SPM, DAG and Cer in pSS patients.

As with UWS, 26 individual SPM species with total fatty acid chain length ranging between C32 and C44 were identified, of which six species were saturated.

**Figure 3:**
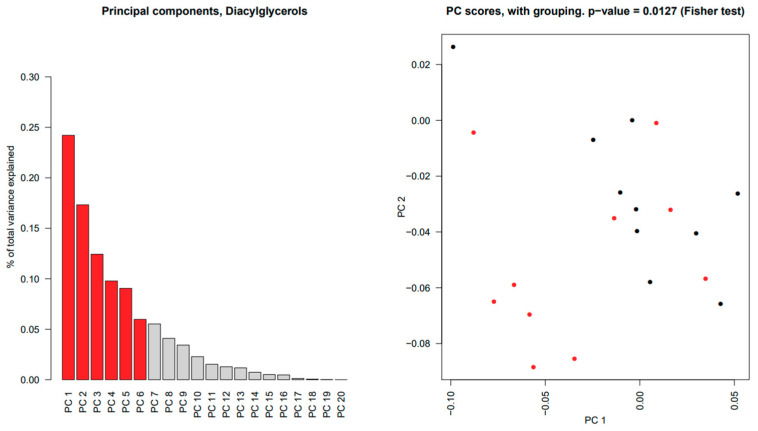
Principal component analysis results of diacylglycerols in stimulated whole saliva. In the (**left panel**), a bar plot of the percentage of the total data variation explained by each estimated principal component; the first 6 principal components (in red) jointly explain 70% of the total data variability. In the (**right panel**), a scatterplot of the principal component scores corresponding to the first two principal components, for primary Sjögren’s syndrome (•) and control subjects (•). Principal components analysis showed a significant difference of *p* = 0.0127.

Several significant differences between the two study groups were found in SWS among individual lipids in Cer, DAG, PC and SPM. Principal component analysis and subsequent multivariate testing showed significant differences in all of these lipid classes (*p* ≈ 0.01 for Cer and DAG, *p* < 0.01 for PC and SPM), as illustrated for DAG in Figure 3; remaining lipid distribution plots for SWS can be found in Supplementary Materials as Supplementary Figures S9–S16. No WE or CE were detected in our analysis.

### 2.4. Triacylglycerols Are Increased While Diacylglycerophosphoinositol and Sphingomyelins Are Decreased in Salivary Glands from pSS Patients

**Figure 4:**
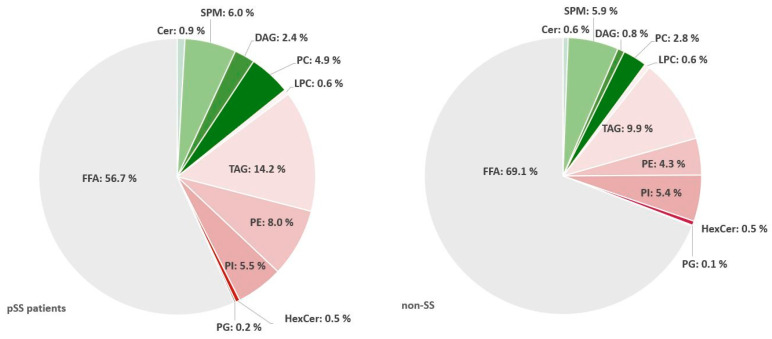
Total distribution of lipid concentration in biopsies. pSS: primary Sjögren’s syndrome; nonSS: non-Sjögren’s syndrome; FFA: free fatty acids; Cer: ceramide; SPM: sphingomyelin; PC: diacylglycerophosphocholine; DAG: diacylglycerol; LPC: lysophosphatidylcholine; TAG: triacylglycerol; PE: diacylglycerophosphoethanolamine; PI: diacylglycerophosphoinositol; HexCer: hexosylceramides; PG: phosphatidylglycerol. Significant differences in individual lipids between glands from pSS patients compared to non-SS sicca subjects were observed in PI, SPM (both *p* < 0.05), and TAG (*p* < 0.01).

Significant differences in individual lipids between glands from pSS patients compared to non-SS sicca subjects were observed in diacylglycerophosphoinositol (PI), SPM, and triacylglycerol (TAG), as identified by t-test. The lipid distribution found in salivary gland biopsies is illustrated in Figure 4; remaining lipid distribution plots for salivary gland biopsies can be found in Supplementary Materials as Supplementary Figures S17–S36. Specifically, PI(C34:0) and SPM(C42:2) were decreased in pSS (both *p* < 0.05), while TAG(C51:2)a was increased in pSS (*p* < 0.01). No significant differences were found between pSS and non-SS control glands concerning Cer, DAG, FFA, hexosylceramides (HexCer), lysophosphatidylcholines (LPC), PC or diacylglycerophosphoethanolamine (PE). Moreover, further statistical analysis by multivariate testing performed on the principal components confirmed no significant differences between groups.

### 2.5. Significant Changes in Individual Species of Diacylglycerophosphocholine, Lysophosphatidylcholine, Sphingomyelins and Wax Esters in Tear Fluid from pSS Patients

**Figure 5:**
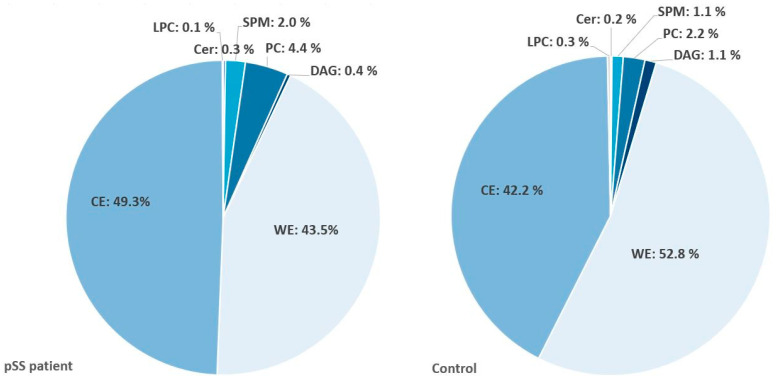
Lipid composition of tears. pSS: primary Sjögren’s syndrome; Cer: ceramide; SPM: sphingomyelin; PC: diacylglycerophosphocholine; DAG: diacylglycerol; WE: wax ester; CE: cholesterol ester; LPC: lysophosphatidylcholine. Significant changes between pSS patients and controls in individual species of PC, LPC, SPM and WE were found (*p* < 0.01 for PC, *p* < 0.05 for the remaining).

In healthy controls, an average of 21.2 µg of lipids was detected in samples, while an average of 47.8 µg of lipids was detected in pSS patients (*p* = 0.32). In both groups, the lipid classes of CE, WE and PC constituted the major components of the lipids in tear fluid (Figure 5).

Significant differences between pSS patients and healthy controls were found in the following individual lipid species: PC(C34:2)a and PC(C36:3)a were increased in pSS (both *p* < 0.01); LPC(C16:0)b was decreased while LPC(C18:1)a was increased in pSS (both *p* < 0.05); SPM(C34:1)a was decreased in pSS (*p* < 0.05); WE(C45:2)a and WE(C47:2)a were increased in pSS (both *p* < 0.05). The distribution of individual PC species as an example is represented in Figure 6; the remaining lipid distribution plots concerning the tear film can be found in Supplementary Materials as Supplementary Figures S37–S52.

**Figure 7:**
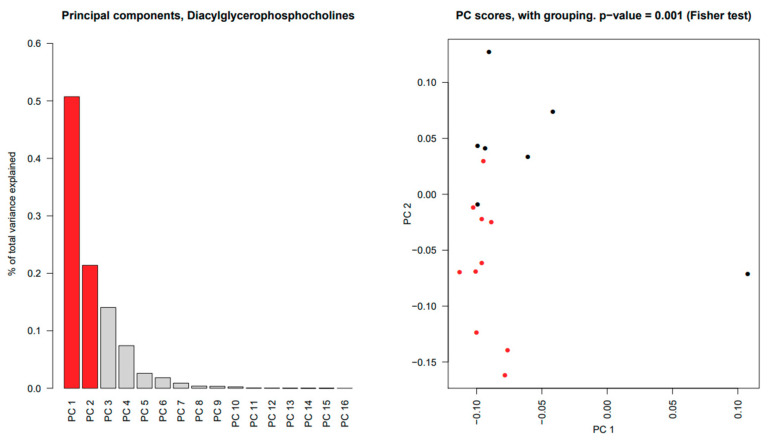
Principal component analysis results of diacylglycerophosphocholines in tears. In the (**left panel**), a bar plot of the percentage of the total data variation explained by each estimated principal component. The first and second principal components (in red) account for the greatest and second greatest variability, 50.7% and 21.4%, respectively of the total data variability. In the (**right panel**), a scatterplot of the principal component scores corresponding to the first two principal components for pSS (•) and control subjects (•). There was a significant difference in the composition of diacylglycerophosphocholines between pSS patients and control subjects (*p* = 0.001).

Principal component analysis and subsequent multivariate Fisher tests showed a significant difference in the composition of PC between pSS patients and control subjects (*p* = 0.001 (Figure 7). LPC reached borderline significance with a *p*-value of 0.07. There were no major differences in the overall distribution of nonpolar lipids between pSS patients and control subjects (*p* > 0.05).

In total, 16 PC species were detected, with PC(C34:1) and PC(C34:2) being the most abundant species. The relative abundance of PC(C34:2 and PC(C36:3) was higher in pSS patients. Ten species of LPC were detected with LPC(C16:0) and LPC(C18:0) being the most predominant species. The relative abundance of LPC(C16:0)b was lower in pSS patients while LPC(C18:1)a was higher in pSS patients. Among the 11 species of SPM, SPM(C34:1)a was the most abundant, and pSS patients had higher relative abundance than controls. The class of (O-acyl)-ω-hydroxy fatty acids (OAHFA) consisted of 23 distinct species, with OAHFA(C48:2), OAHFA(C50:2), and OAHFA(C52:2) as the three species found in highest relative abundance. Species of WE in tears were predominantly unsaturated fatty acyl moieties with total fatty acid chain length from C37 to C48. There was a tendency of higher levels of medium to high molecular weight WE in pSS patients than control subjects. At least 23 different species of CE were observed, with total fatty acid chain length ranging from C16 to C26. The most abundant compounds of the CE family were CE(C18:1); CE(C18:2), CE(C22:1), CE(C24:0), CE(C24:1) and CE(C26:1). Nonetheless, no significant differences in relative abundance of individual species in CE were observed.

## 3. Discussion

The current study used the HPLC/MS-based approach for a comprehensive, qualitative and comparative characterization of the lipids in human saliva and tears, as well as biopsies of labial salivary glands from pSS patients. Our analyses demonstrated the complexities of the human saliva and tear fluid lipidome and showed significant differences in lipid profiles between pSS patients and control subjects in the aforementioned materials.

**Figure 8:**
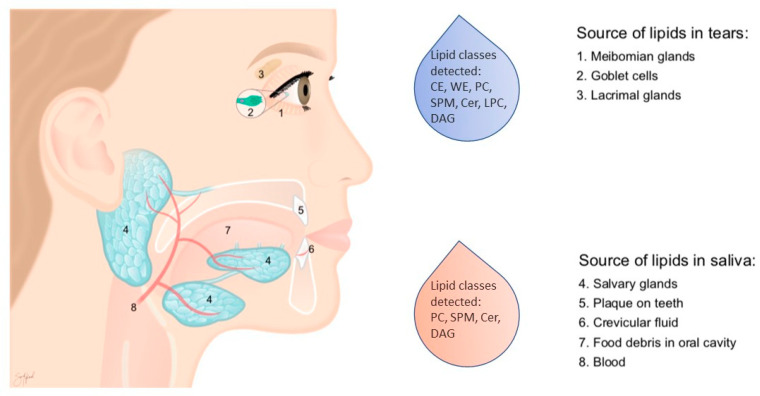
An overview of identified lipids and their origin. CE: cholesterol ester; WE: wax ester; SPM: sphingomyelin; Cer: ceramide; LPC: lysophosphatidylcholine; DAG: diacylglycerol; PC: diacylglycerophosphocholines. (Figure produced by Sara Nøland).

In both pSS patients and healthy controls, the classes of SPM, PC, and DAG were the major lipid constituents of resting and stimulated whole saliva. In contrast, CE, WE and DAG constituted the major components of the tear fluid lipidome. These differences reflect the sources and functions of lipids in saliva and tears, respectively. A summary of identified lipids and their origin is illustrated in Figure 8.

In the present study, principal component analysis of salivary lipids and subsequent multivariate testing revealed significant differences between pSS patients and healthy controls in every investigated lipid class. Furthermore, a higher level of total saliva lipid concentration was detected in pSS patients vs. healthy controls, a finding corroborated by Slomiany et al. [22]. However, their study was based on stimulated parotid saliva, while the present study examined whole saliva [22]. Additionally, Larsson et al. reported comparable total lipid concentration values in healthy adults in stimulated saliva from parotid, submandibular and whole saliva [18]. Theoretically, the increased lipid concentration in pSS patients could be caused by decreased fluid volume due to hyposalivation, increased lipid secretion or a combination of these. Hyposalivation is a well-established hallmark of SS, and we acknowledge that this most likely is the primary contributing factor. Furthermore, other possible sources of lipids in general, and polar lipids specifically, in the oral cavity are cell membranes that consist of various, mainly amphiphilic, lipid species, and sloughing from both eukaryotic and prokaryotic cells [34].

Previous studies have indicated that between 50–99% of parotid and submandibular lipids are non-polar, and that saliva collected from healthy subjects mainly consisted of CE, WE, FFA and squalene [16,17,18,22]. In the present study, the lipid composition of UWS and SWS among healthy controls consisted mainly of polar lipids, with no WE or CE detected. One possible explanation for this discrepancy might be differences in the type of saliva collected. As opposed to the present work, two of the aforementioned studies analyzed whole saliva [16,18], while the rest used parotid or submandibular saliva [17,22]. Since it has been shown that the labial salivary glands secrete a higher degree of polar lipids, an increased proportion of polar lipids might be expected when analyzing whole saliva [20].

Another relevant source of lipid profile discrepancy could be differences in methodology used for lipidomic analysis. The abovementioned studies used thin layer chromatography, as opposed to the much more accurate HPLC/MS employed in the current study [16,17,18,22]. As far as we can tell, the present study is the first to examine the lipid profile of human saliva employing MS. Differences in study population might also have contributed to the inconsistency, as variability in inter-subject oral lipase activity has been described, with lipolytic activity found in some, but not all study participants [35]. Also, the individual oral microbiome might be affected by FFA released by lingual lipase-driven degradation of oral lipids [36]. Whether this lipolytic activity is caused by endogenously secreted lipases within the oral cavity, reflux of gastric secretions or intestinal enzymes, or from microbially produced enzymes in the oral cavity remains unknown. Nonetheless, inter-subject variability in oral lipolytic activity may influence the presence and detectability of oral lipids between subjects. Interestingly, a recent study utilizing MS to analyze the lipidomic profile of dental plaques identified mainly polar lipids, with about 50% of total lipids consisting of TAG and PC [37]. These findings present yet another possible source of polar lipids within the oral cavity.

Slomiany et al. revealed twice as many total lipids as well as three times as many glycolipids and 20 times more phospholipids in pSS patients vs. healthy controls when assessing the lipid composition of parotid saliva [22]. Specifically, they described a significant increase in both PC and SPM in pSS patients. This result is in accordance with our findings in whole saliva. Contrary to their observations, however, we found a decrease of DAG in pSS patients. The cause of this discrepancy may be due to our analysis of whole saliva while Slomiany et al. examined parotid secretions, or due to differences in analytic methodology, as discussed above.

As previous studies have revealed an increased proportion of adipose tissue as well as an upregulation of genes responsible for adipocyte development in minor salivary glands of pSS patients, we aimed to explore the lipidomic profiles of salivary glands from pSS patients and non-SS sicca controls [10,11]. Several significant differences were detected.

Specifically, the polar lipids PI(C34:0) and SPM(C42:2)a were decreased and the nonpolar lipid TAG(C51:2)a was increased in patients diagnosed with pSS. Interestingly, SPM(C42:2)a was also found to be significantly increased in SWS of pSS patients, with no difference in UWS. Neither PI nor TAG were detected in either UWS or SWS. As far as we can discern, this is the first study to examine the lipidomic profile of minor salivary glands, both alone and in combination with analyses of whole saliva. The clinical relevance of these differences is so far unknown but may point towards a link between SPM(C42:2)a in salivary glands and stimulated whole saliva. Our findings warrant further investigation.

In the current study, lipidomic analysis of tear fluid from pSS patients and healthy controls detected several polar and nonpolar lipid classes consistent with previous reports [31,32,38,39]. Significant changes in individual species of PC, LPC, SPM and WE were observed in tear fluid from pSS patients compared to healthy controls.

In line with previous studies, the polar lipids PC, LPC, SPM, Cer and OAHFA were detected in our samples [38,40]. Polar lipids are likely to originate from conjunctiva, cornea and tears produced by the lacrimal glands [41,42,43]. Multivariate tools, such as principal component analysis, allow detection of subtle changes in the lipidome that may be associated with factors such as age, sex and underlying diseases [44,45]. In the present study, multivariate Fisher test showed that the profiles of lipid species in PC among pSS patients differed from that of control subjects, which might be caused by disease-related damage to the lacrimal gland and ocular surface. Whether these lipidomic alterations might serve as a potential marker for dry eye disease generally or pSS specifically is an intriguing concept in want of further research.

In accordance with previous reports, the meibum-associated CE and WE were the predominant nonpolar lipid classes, whereas phospholipids comprised a substantial part of the polar lipids in our study [31,32,38,39]. However, there can be significant inter-individual variability of the lipid profile of human tear fluid [44]. For instance, our data showed that the relative abundance of WE ranged from about 30% to 56% in pSS patients, and from 37% to 73% in control subjects. The WE species detected in the highest abundance included WE(C42:2), WE(C43:1) and WE(C44:1), which is in accordance with findings by Brown et al. [31]. Moreover, our data showed a trend towards higher levels of medium to high molecular weight WE in pSS patients than control subjects. A reduction in WE of low molecular mass could possibly lead to decreased tear film stability, as low molecular mass WE have relatively high polarity and might serve as transitional lipids in bridging the interaction between the amphiphilic lipid sublayer with the other nonpolar lipids [43]. Polar lipids, on the other hand, are believed to create tear film stability by forming a stable interaction between nonpolar lipids and the polar aqueous-mucin phase of the tear film. The most abundant CE in the current study were CE(C18:1)a, CE(C22:1)a and CE(C24:1)a, while Brown et al. reported CE(C24:0), CE(C25:0) and CE(C26:0) in the highest abundance [31]. Different analytical techniques and inter-individual variation may explain the differences. These findings may indicate that substitution therapy in the form of eye drops with non-polar lipids of low molecular mass might help to alleviate symptoms of dry eye.

Differences in tear collection procedures (by microcapillary tube with or without flush or Schirmer strips) and inherent intra- and inter-individual variability may contribute to variations in the amount and composition of tear lipids. Borchman et al. compared the tip portion of the Schirmer strip, which was in direct contact with the lid margin, to the wetted portion, which was not [40]. They found that the lipids extracted from these two sources were similar and estimated that meibum lipids comprise less than 5% of the tear lipids from the tip of the Schirmer strip. Similarly, Lam and associates demonstrated that the lipid profiles in tear fluid collected using Schirmer strips were similar to those collected using microcapillary tubes with or without a saline flush, while the Schirmer strips captured greater absolute amounts of tear lipids [38]. Moreover, they also reported high reproducibility in lipid composition across samples, even for those with very low wetted length, indicating that the method is applicable even to patients with severe dry eye disease [38]. As previous studies have shown a low degree of contamination as well as a high degree of lipid accumulation, it is likely that the lipidomic profile found using Schirmer strips in the current study is representative of lipids in the tears.

In the present study, we revealed several significant differences in the lipidomic profiles of saliva and tears in human patients suffering from pSS compared to healthy controls. Specifically, we demonstrated disparities in all identified salivary lipid classes, as well as an overall increased concentration of salivary lipids among pSS patients. Moreover, differences in individual lipid species from glandular biopsies corresponding to findings in stimulated saliva represent an intriguing avenue for further research. Further, in tear fluid we found variations in several individual polar and non-polar lipid species, and significant differences in the PC lipid class as a whole. These differences in the lipidomic composition of saliva and tears in patients suffering from pSS may lay the foundation for future in-house clinical tests diagnosing pSS through either cut-offs regarding lipid concentration or recognition of specific lipidomic profiles.

In conclusion, despite a relatively small sample size, we were able to identify several significant changes between the groups. This highlights the power of lipidomic analyses in saliva and tears. The identified differences further our understanding of the pathogenesis of xerostomia and xerophthalmia in pSS. This and other follow-up studies may represent an initial step towards identifying novel diagnostics and therapeutics in this debilitating disease.

## 4. Materials and Methods

### 4.1. Study Participants

Ten saliva and tear samples from female patients diagnosed with pSS according to the 2002 AECG classification criteria [1] and ten saliva and tear samples from age- and gender-matched controls were included in this study. Additionally, biopsies from three female patients diagnosed with pSS and three seronegative female patients with sicca symptoms who did not meet the AECG criteria of pSS (non-SS sicca subjects) were analyzed. All patients were examined by rheumatologists prior to the study. Clinical characteristics according to the AECG criteria of these patients are summarized in Supplementary Table S1. As previous metabolomic studies have reported significant gender differences, all included study participant were female [34,46].

Healthy age- and sex-matched controls without symptoms of dryness from eyes and mouth were recruited locally. Written informed consent was acquired from all subjects and the study was approved by the Regional Medical Ethics Committee of South-East Norway (2015/363; approval date 29 April 2015).

Patients and healthy controls were examined at the Dry Mouth Clinic at the Faculty of Dentistry, University of Oslo, and the Norwegian Dry Eye Clinic, Oslo, Norway.

### 4.2. Saliva Collection

Participants were encouraged to avoid any food or drink, oral hygiene measures and smoking for 1 h prior to examination. Following a comprehensive oral examination at the Dry Mouth Clinic, UWS and SWS were collected from all subjects as previously described [8]. In short, standardized sialometry was performed on all participants, yielding saliva secretion rates for UWS and SWS. UWS was gathered for 15 min in ice chilled, pre-weighed plastic cups. Subsequently participants chewed on a paraffin wax tablet (paraffin pellets, Ivoclar Vivadent, Shaen, Lichtenstein) for 30 s while swallowing any saliva in the mouth. SWS was collected for 5 min under continued chewing in a new ice chilled, pre-weighed plastic cup. Finally, saliva samples were weighed, and secretion rates calculated for UWS and SWS (g/mL = mL/min).

### 4.3. Biopsies of Labial Salivary Glands

Biopsies of lower labial salivary glands were obtained at the Department of Oral Surgery and Oral Medicine, University of Oslo, Oslo, Norway. Through a superficial horizontal incision to the left of the inner aspect of the lower lip’s midline, 5–10 glands were bluntly dissected. All but one gland were sent for histological evaluation, while one gland was placed in RNAlater and stored at −150 °C until analyzed.

### 4.4. Tear Fluid Collection

Following a thorough examination of the ocular surface at the Norwegian Dry Eye Clinic, tear fluid was collected from both eyes as previously described [8]. Tears were collected by placing a Schirmer test strip (HAAG-STREIT, Essex, UK) on the inside of the lower eyelid of each eye for a minimum of 5 min at room temperature. The Schirmer strips were subsequently added to 0.5 mL of 0.1 μm filtered phosphate-buffered saline (PBS) (Gibco, pH 7.4, ThermoFisher Scientific, Oslo, Norway) and stored at −80 °C.

### 4.5. Lipid Extraction and Quantification

Saliva and Schirmer paper samples from 20 female participants were prepared as follows: for saliva an aliquot of 100 µL sample was used while for tear fluid the Schirmer paper was allowed to dry prior to cutting the paper in smaller sections. Then isopropanol (300 µL for saliva; 100 µL for tears) containing lipid internal standards at about 1 µg/mL was added to each of the samples. The solution was thoroughly vortexed prior to centrifugation at 4000 rpm at 10 °C for 15 min. The supernatant was then transferred to a high-performance liquid chromatography (HPLC) vial containing a 500 µL HPLC insert and subsequently analyzed on the mass spectrometry-based lipidomic platform.

Lipidomic analysis of the salivary gland biopsies was performed as follows. Each HPLC vial was weighed before and after the biopsy sample was added (approx. 5 mg). Thereafter, 300 µL of Folch extraction solution containing internal standards with a concentration ranging from 1 to 7 µg/mL was added to the vials. The samples were homogenized using a handheld homogenizer (VWR VDI12). After homogenization, 50 µL of water was added to the samples and they were vortexed prior to centrifugation at 4000 rpm for 15 min at 10 °C. An aliquot of 50 µL of the lower organic phase was diluted with 100 µL isopropanol and was subsequently analyzed on the lipidomic platform as described below.

### 4.6. Untargeted Comprehensive Lipidomic Analysis

Untargeted comprehensive lipidomic analysis was performed on saliva, minor salivary glands and tear fluid isolated from Schirmer paper using HPLC coupled to time-of-flight mass spectrometry (TOF-MS). The platform allows determination of glycerolipids, glycerophospholipids, cardiolipins, sphingolipids, free fatty acids, free cholesterol, cholesterol esters, wax esters, diesters and OAHFA, as described in previously published work [47,48]. In total, 86, 146 and 132 specific lipids within these classes were identified in, saliva, minor salivary glands and tears, respectively. A 1260 Agilent chromatographic system comprising an autosampler, a binary pump and a TCC column heater unit coupled to a TOF-MS with Agilent JetStream ionization module for enhanced sensitivity was used and operated in both positive and negative ionization mode, as previously described [47,48]. To obtain high-resolution chromatographic separation of the lipids, a C18-EVO Kinetex analytical column (column dimensions 2.1 × 150 mm, 2.6 µm) was used with a flow rate of 0.7 mL/min.

Lipids were separated using a mobile phase gradient. The mobile phases used were as follows (volume ratio, constituents): mobile phase A (47.5:17.5:35 *v*/*v*,15 mmol/L ammonium formate, acetonitrile, isopropanol) and mobile phase B (70:25:5 *v*/*v*, isopropanol: acetonitrile, 15 mM ammonium formate). The gradient used was as follows (time in min, % mobile phase B): 0 min (0% B), 12 min (40% B), 33 min (85% B), 33.1 min (100% B) 38.5 (100% B) and 38.6 min (0% B). Injected volume was 4 µL (positive mode) and 9 µL (negative mode).

Current instrumentation using TOF-MS is not able to distinguish between isobaric compounds (i.e., those having identical molecular masses). However, isobaric structural isomers may be separated chromatographically and are hence denoted by a suffix (a, b or c)—e.g., the diacylglycerol species DAG(36:2)a and DAG(36:2)b.

Individual lipid species were quantified by referencing to spiked internal standards with concentrations from 1–5 µg/mL. One single non-endogenous internal standard was used per lipid class investigated. There were no commercially available lipids suitable as an internal standard for OAHFA; thus, quantification of the OAHFA lipid group was not possible and relative abundancies for the individual species within this lipid class was calculated.

### 4.7. Statistical Analysis

Statistical analysis was performed using R software for statistical computing, version 4.0.2 [49]. First, differences in average concentrations and total lipid amounts were analyzed using the Mann–Whitney U test. Secondly, univariate analyses were carried out on all compounds within each lipid group: independent samples t-tests were used to test the difference in mean between pSS patients and controls when data were normally distributed; otherwise, Wilcoxon tests were applied. Thirdly, all lipid groups were analyzed using principal component analysis to estimate the most relevant principal components (i.e., linear combinations of the compounds) for explaining the variation in the data. Following a standard approach, the first k principal components explaining at least 70% of the total data variation were considered relevant and included in subsequent analyses [50]. The multivariate Fisher test was then applied to the relevant components for testing the difference between pSS patients and controls, with a low p-value indicating a joint contribution of the compounds in demonstrating a difference in the mean of relative abundance between groups. A scatterplot of the first two principal components’ scores, i.e., the data projections on the subspace identified by the first two principal components, was used to display the capability of the principal components to capture the variability between pSS patients and controls.

## Figures and Tables

**Figure 6:**
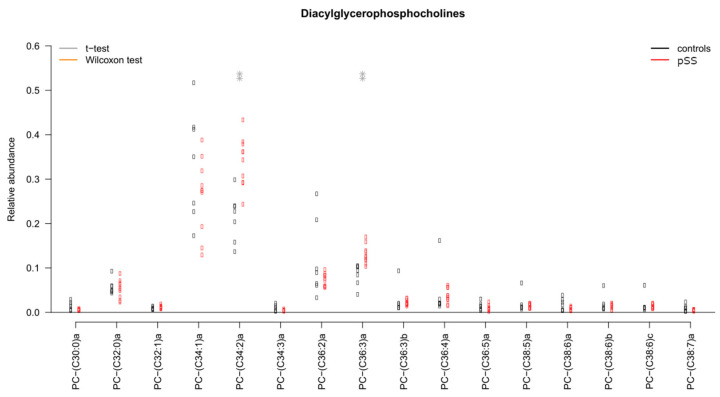
Distribution of individual diacylglycerophosphocholines in tears. ** *p* < 0.01; pSS: primary Sjögren’s syndrome. Significant chances were found between pSS patients and controls in PC(C34:2)a and PC(C36:3)a.

## Data Availability

All data are contained within the manuscript and supplementary materials.

## Supplementary Materials

Supplementary materials can be found at https://www.mdpi.com/article/10.3390/ijms22168997/s1.
