# Supplementary material for: Characterization of Lipids in Saliva, Tears and Minor Salivary Glands of Sjögren’s Syndrome Patients Using an HPLC/MS-Based Approach
Source: Int J Mol Sci. 2021 Aug 20;22(16):8997. doi: 10.3390/ijms22168997 (PMC8396590; doi:10.3390/ijms22168997)
Supplement: Supplementary file 1 [file ijms-22-08997-s001.zip › ijms-1297373-supplementary.pdf]

Supplementary Materials

Characterization of Lipids in Saliva, Tears and Minor Salivary Glands of Sjögren's Syndrome Patients Using an HPLC/MS-Based Approach

Fredrik Fineide [1,2], Xiangjun Chen [3], Thomas Bjellaas [4], Valeria Vitelli [5], Tor Paaske Utheim [1,2,6,7], Janicke Liaaen Jensen [3] and Hilde Kanli Galtung [7,*]

1 Department of Plastic and Reconstructive Surgery, Oslo University Hospital, 1171 Oslo, Norway; fre_fin@hotmail.com (F.F.); uxutto@ous-hf.no (T.P.U.)
2 The Norwegian Dry Eye Clinic, Ole Vigs Gate 32 E, 0366 Oslo, Norway
3 Department of Oral Surgery and Oral Medicine, Faculty of Dentistry, University of Oslo, 0317 Oslo, Norway; chenxiangjun1101@gmail.com (X.C.); j.c.l.jensen@odont.uio.no (J.L.J.)
4 VITAS Analytical Services, 0349 Oslo, Norway; tbj@vitas.no
5 Department of Biostatistics, Institute of Basic Medical Sciences, Faculty of Medicine, University of Oslo, 0316 Oslo, Norway; valeria.vitelli@medisin.uio.no
6 Department of Medical Biochemistry, Oslo University Hospital, 1171 Oslo, Norway
7 Department of Oral Biology, Faculty of Dentistry, University of Oslo, 0316 Oslo, Norway
* Correspondence: h.k.galtung@odont.uio.no

Supplementary Table S1. Clinical characteristics according to the AECG criteria.

No.	Age (Years)	Dry Mouth[a]	Dry Eyes[a]	Schirmer[b]	Saliva[c]	Anti-SSA[d]	Anti-SSB[d]	FS[e]
pSS1*	48	+	+	+	+	+	+	NT
pSS2**	48	+	+	+	+	+	+	NT
pSS3	57	+	+	+	+	+	+	NT
pSS4	35	+	+	+	+	+	+	NT
pSS5	71	+	+	-	-	+	-	NT
pSS6	47	+	+	+	+	+	+	NT
pSS7*	64	+	+	+	+	+	+	NT
pSS8*†	64	+	+	+	+	+	+	6
pSS9**	54	-	+	+	+	+	-	1
pSS10	72	-	+	+	+	+	-	<1
pSS11**	60	-	-	+	+	+	+	3
pSS12**	64	+	+	+	+	+	-	0
pSS13	68	+	+	-	+	+	+	NT
				Biopsies pSS				
pSS14	59	-	+	+	+	+	-	0
pSS15	71	+	+	-	+	+	-	<1
pSS16	69	-	-	+	+	+	-	2

*Only tears tested; **Only saliva tested; †Schirmer strip of both left and right eye included; pSS: primary Sjögren's syndrome. NT: not tested; FS: Focus score in minor salivary glands; [a] Presence of ocular or oral symptoms. "+" indicates ≥1 confirmative answer to questions from the 2002 AECG criteria, see reference number 1 of main manuscript. [b] Values are in mm/5 minutes; normal flow >5 mm/5 minutes. "+" indicates tear secretion ≤5 mm/5 minutes in at least one eye. [c] Values are in ml/15 minutes; normal flow >1.5 ml/15. "+" indicates unstimulated whole saliva secretion ≤1.5 ml/15 minutes. [d] Autoantibody production was assessed by ELISA. [e] Values are the number of focal infiltrates/4 mm^2 tissue area containing >50 mononuclear cells.

UWS

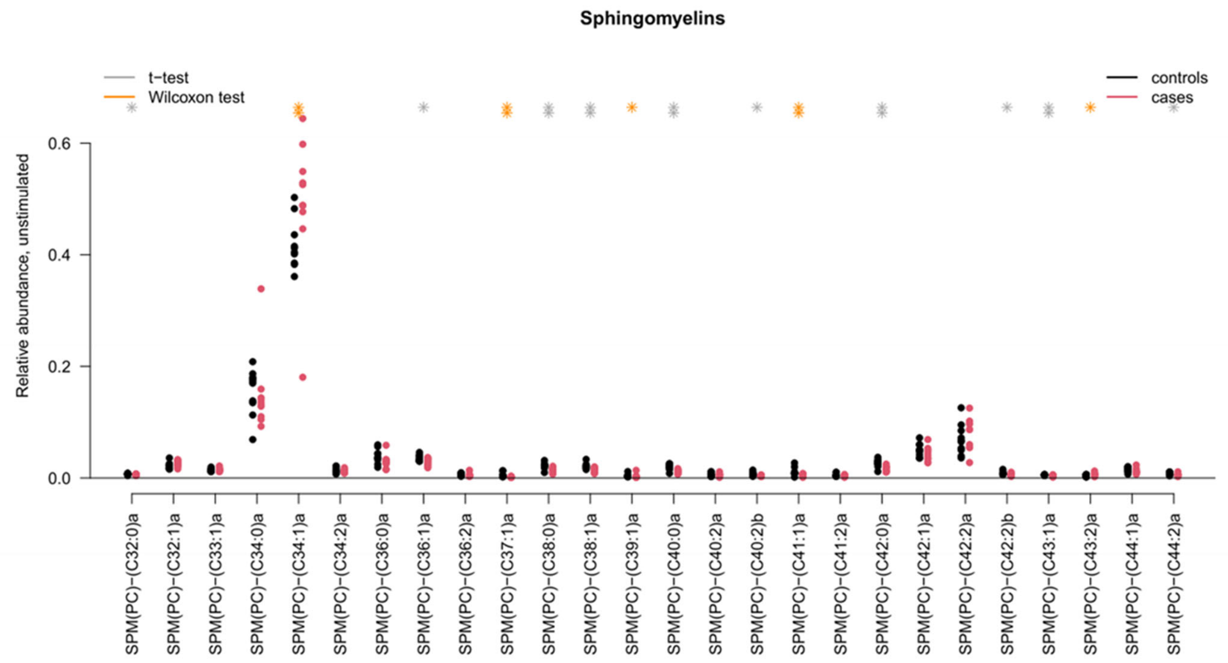

Supplementary Figure S1. Distribution of sphingomyelin in unstimulated saliva. SPM: sphingomyelin. *p<0.05; **p<0.01.

UWS

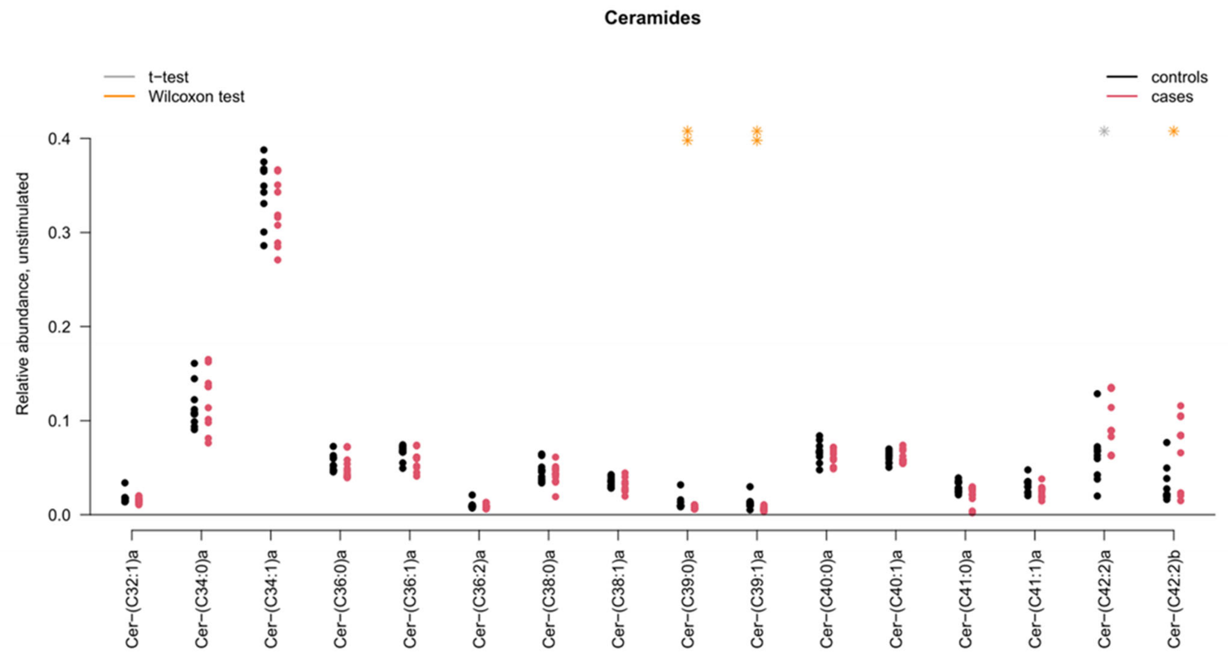

Supplementary Figure S2. Distribution of ceramide in unstimulated saliva. Cer: ceramide. *p<0.05; **p<0.01.

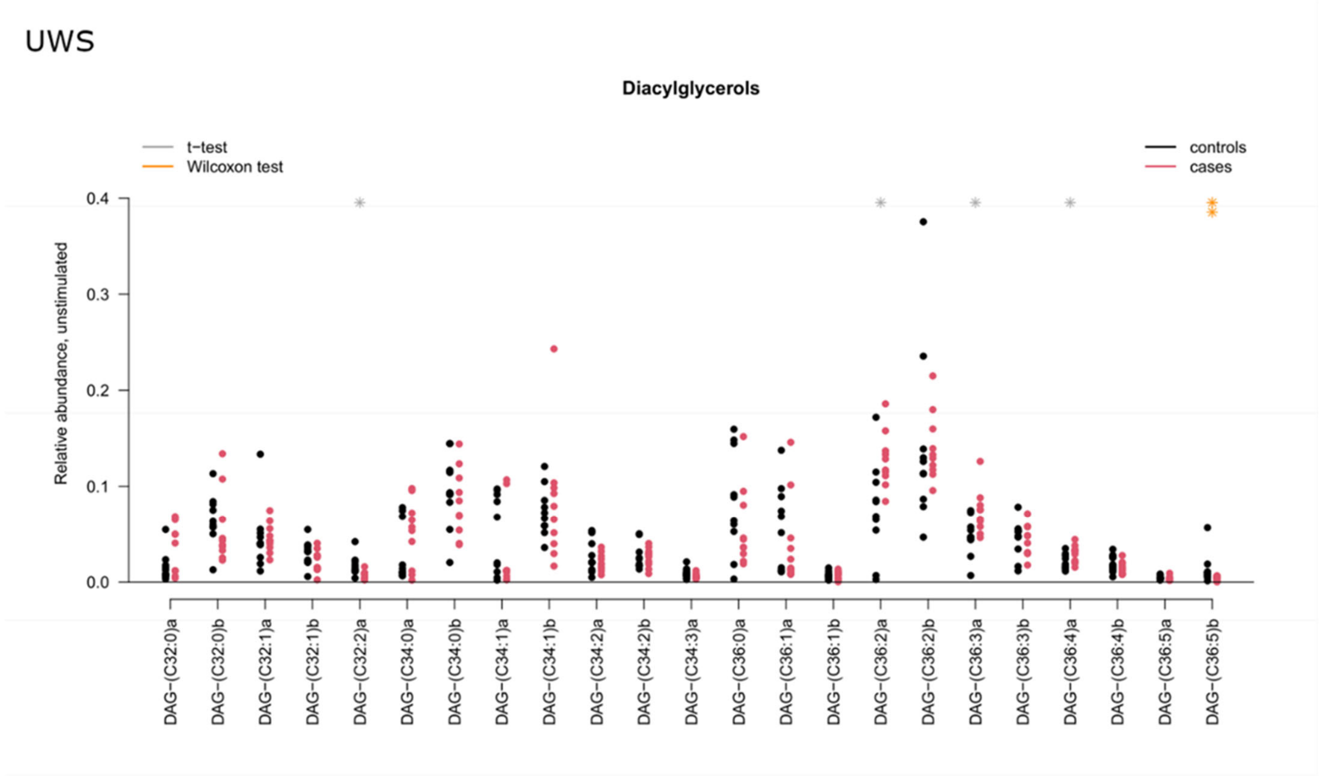

Supplementary Figure S3. Distribution of diacylglycerol in unstimulated saliva. DAG: diacylglycerol. *p<0.05; **p<0.01.

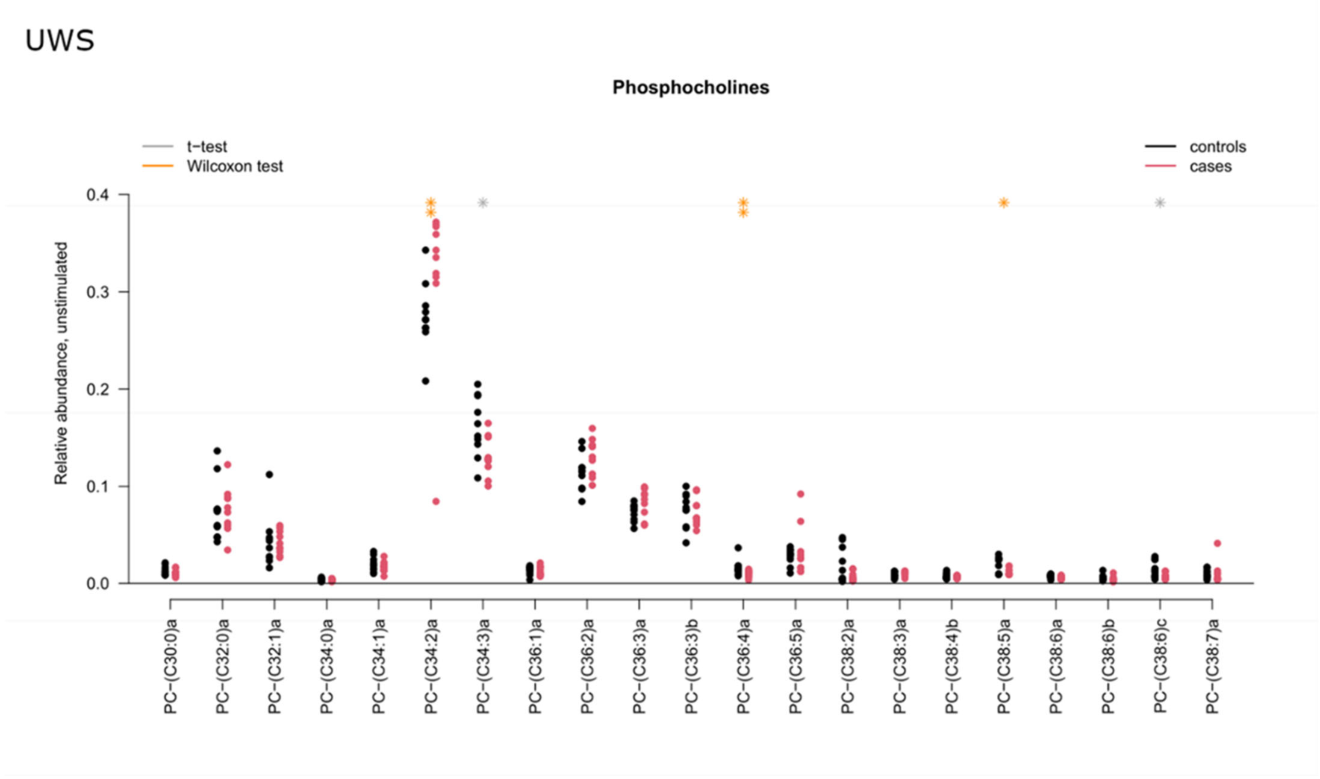

Supplementary Figure S4. Distribution of phosphocholine in unstimulated saliva. PC: phosphocholine. *p<0.05; **p<0.01.

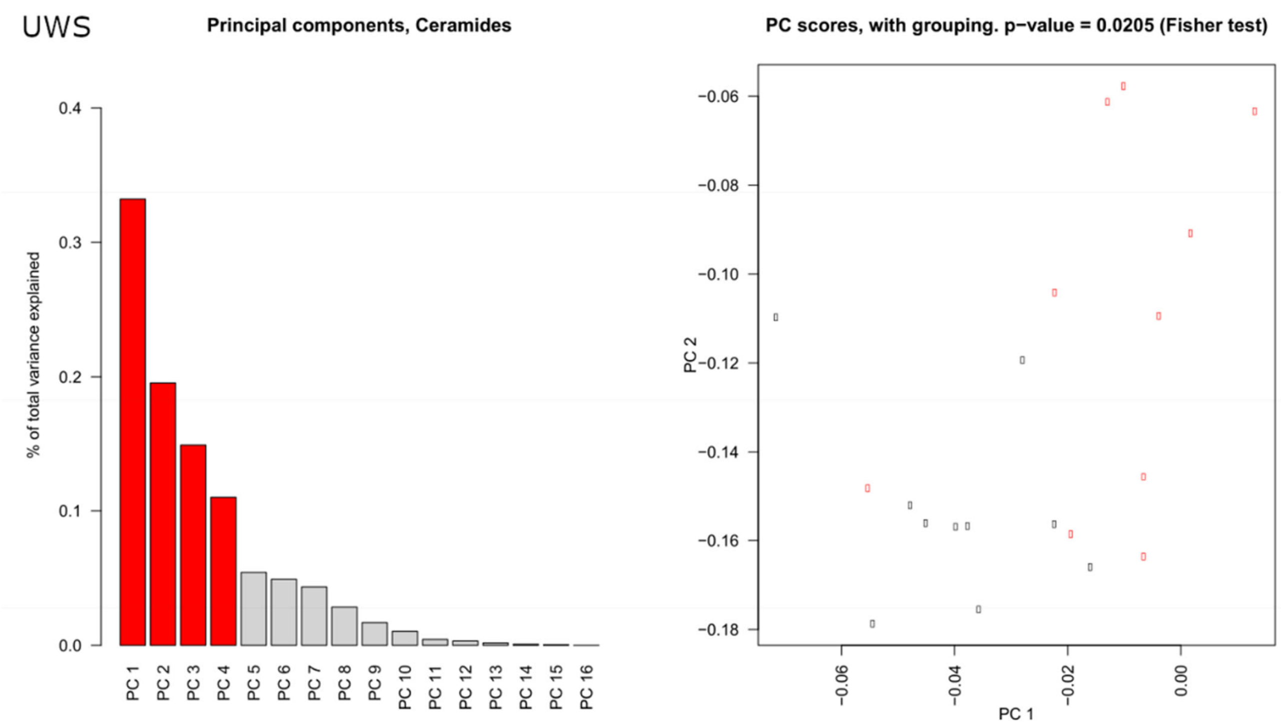

Supplementary Figure S5. Principal component analysis results on ceramides in unstimulated whole saliva. In the left panel, bar plot of the percentage of the total data variation explained by each estimated principal component. In the right panel, scatterplot of the principal component scores for primary Sjögren's syndrome (•) and control subjects (•). PC: principial component. Principal components analysis showed a significant difference of p=0.0205.

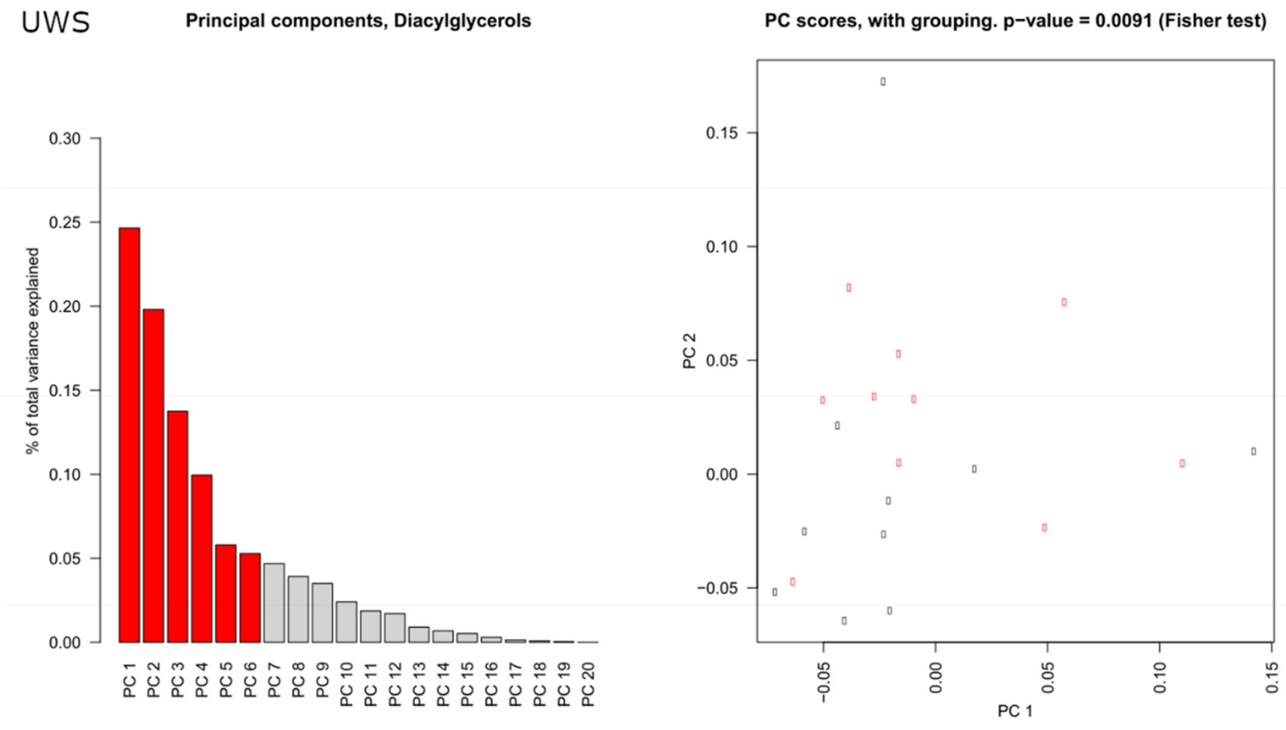

Supplementary Figure S6. Principal component analysis results on diacylglycerol in unstimulated whole saliva. In the left panel, bar plot of the percentage of the total data variation explained by each estimated principal component. In the right panel, scatterplot of the principal component scores for primary Sjögren's syndrome (•) and control subjects (•). PC: principial component. Principal components analysis showed a significant difference of p=0.0091.

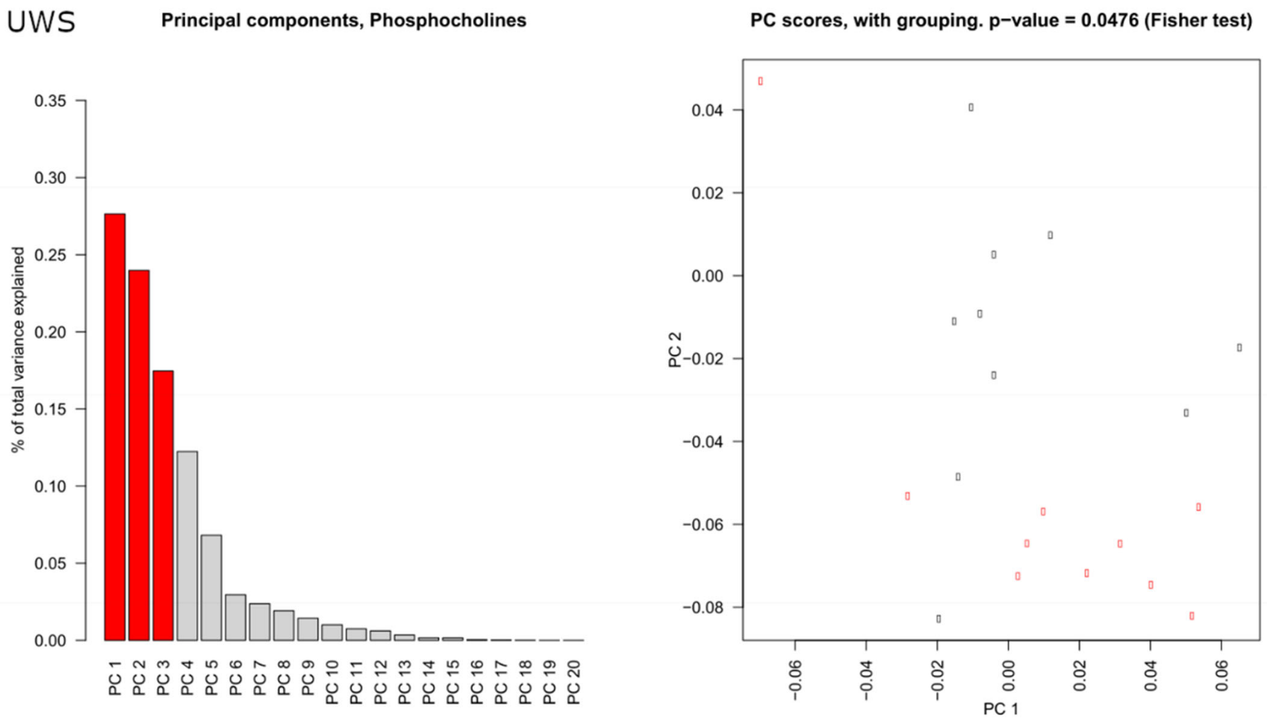

Supplementary Figure S7. Principal component analysis results on phosphocholine in unstimulated whole saliva. In the left panel, bar plot of the percentage of the total data variation explained by each estimated principal component. In the right panel, scatterplot of the principal component scores for primary Sjögren's syndrome (•) and control subjects (•). PC: principial component. Principal components analysis showed a significant difference of p=0.0476.

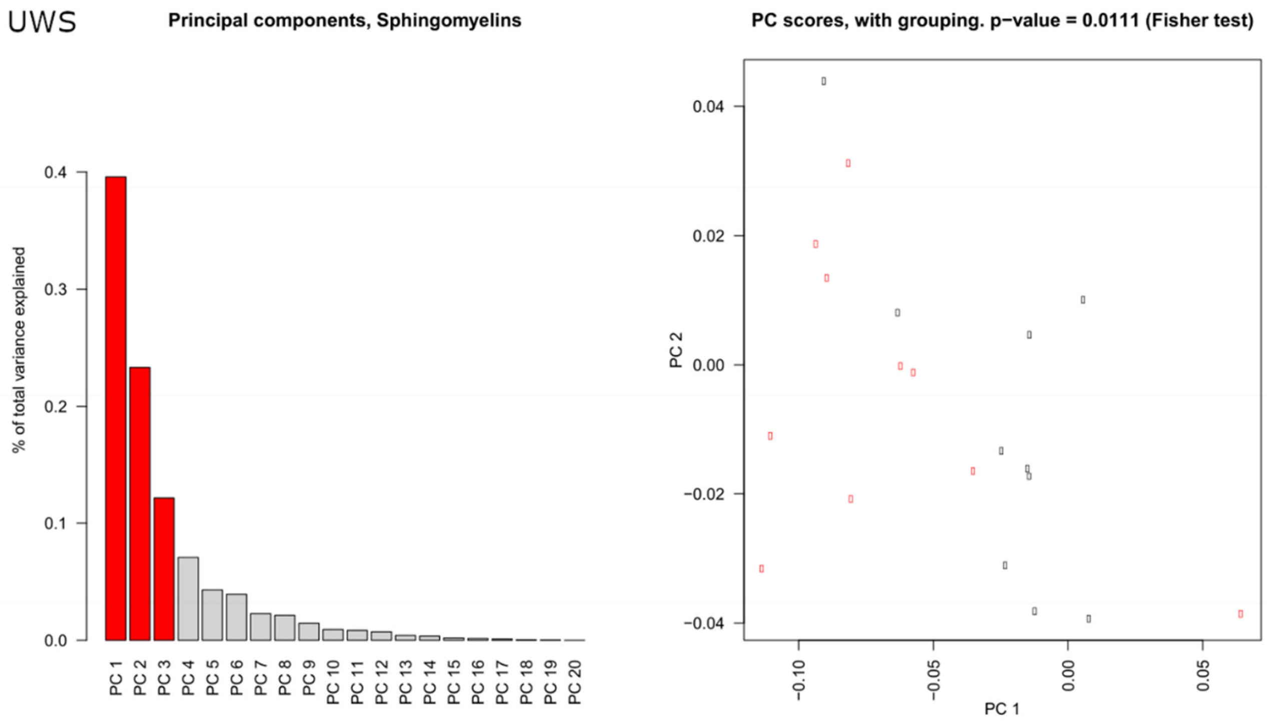

Supplementary Figure S8. Principal component analysis results on sphingomyelin in unstimulated whole saliva. In the left panel, bar plot of the percentage of the total data variation explained by each estimated principal component. In the right panel, scatterplot of the principal component scores for primary Sjögren's syndrome (•) and control subjects (•). PC: principial component. Principal components analysis showed a significant difference of p=0.0111.

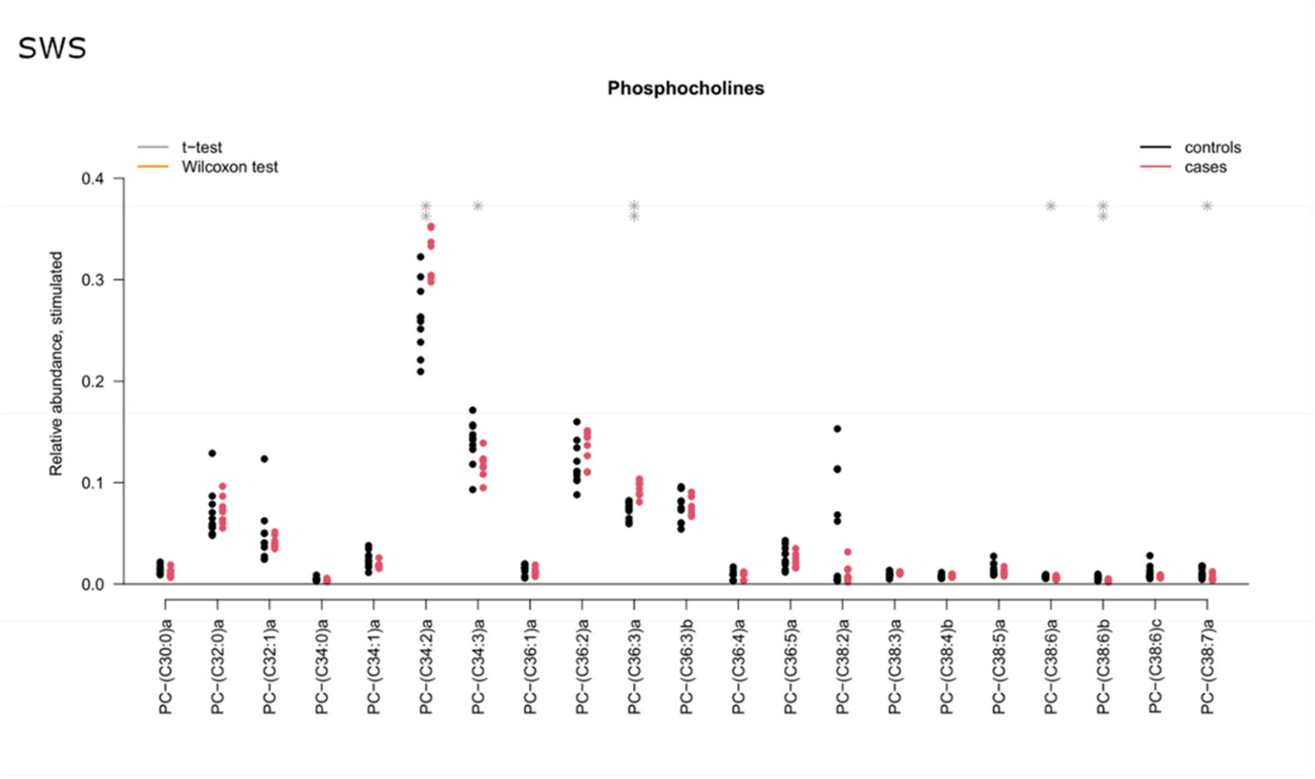

Supplementary Figure S9. Distribution of phosphocholine in stimulated whole saliva. PC: phosphocholine. *p<0.05; **p<0.01.

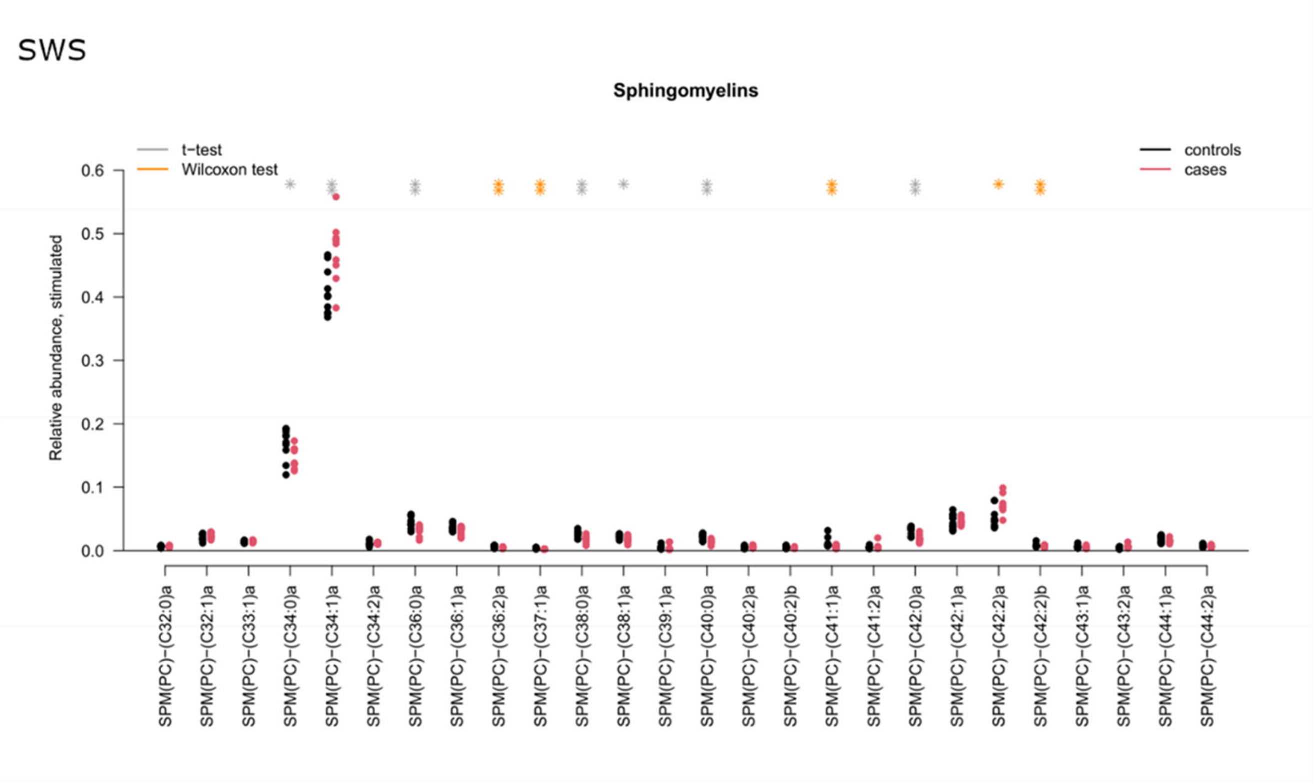

Supplementary Figure S10. Distribution of sphingomyelin in stimulated whole saliva. SPM: sphingomyelin. *p<0.05; **p<0.01.

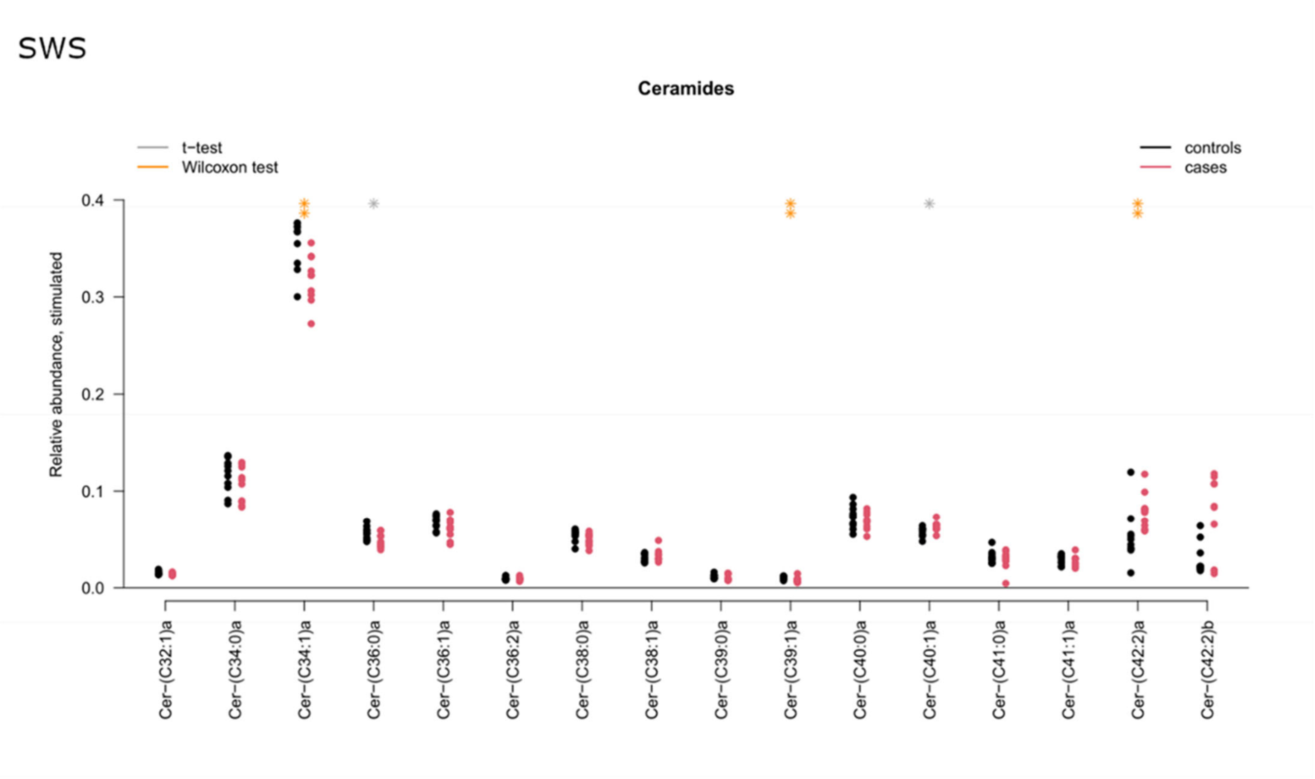

Supplementary Figure S11. Distribution of ceramide in stimulated whole saliva. Cer: ceramide. *p<0.05; **p<0.01.

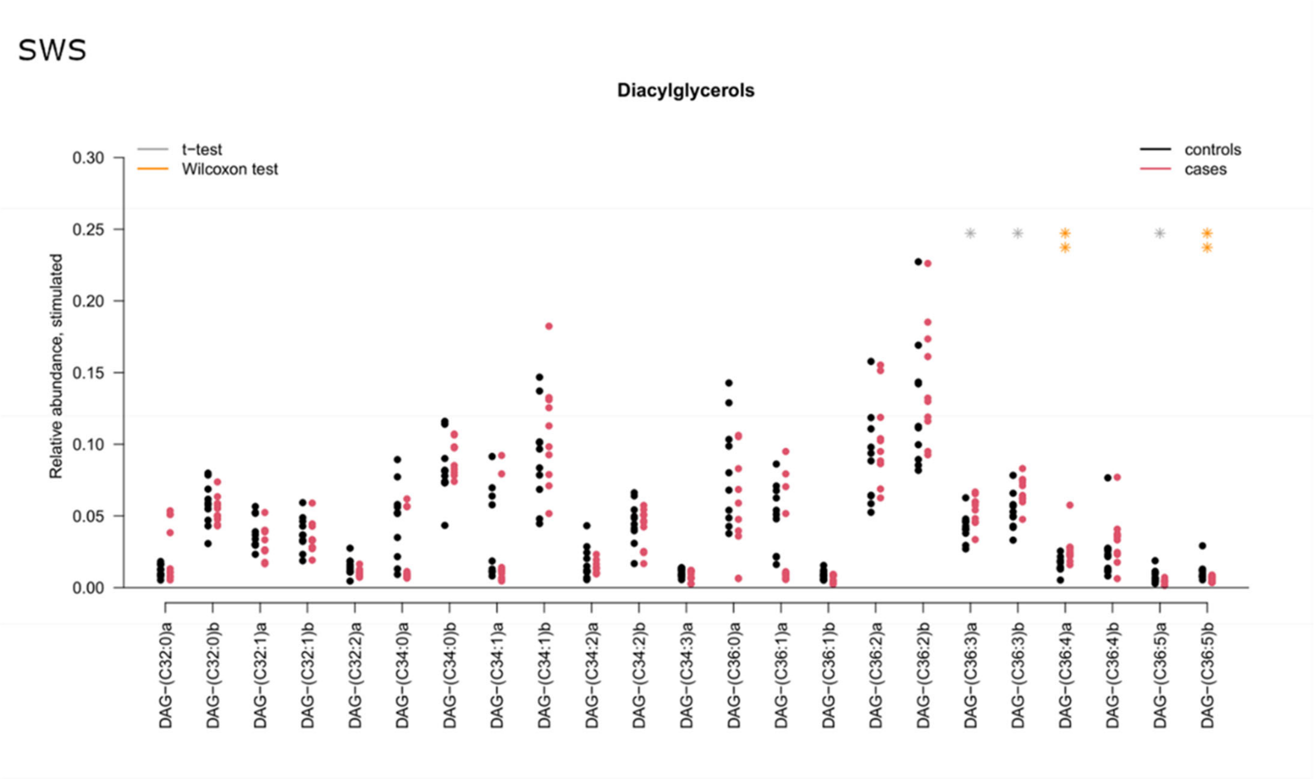

Supplementary Figure S12. Distribution of diacylglycerol in stimulated whole saliva. DAG: diacylglycerol. *p<0.05; **p<0.01.

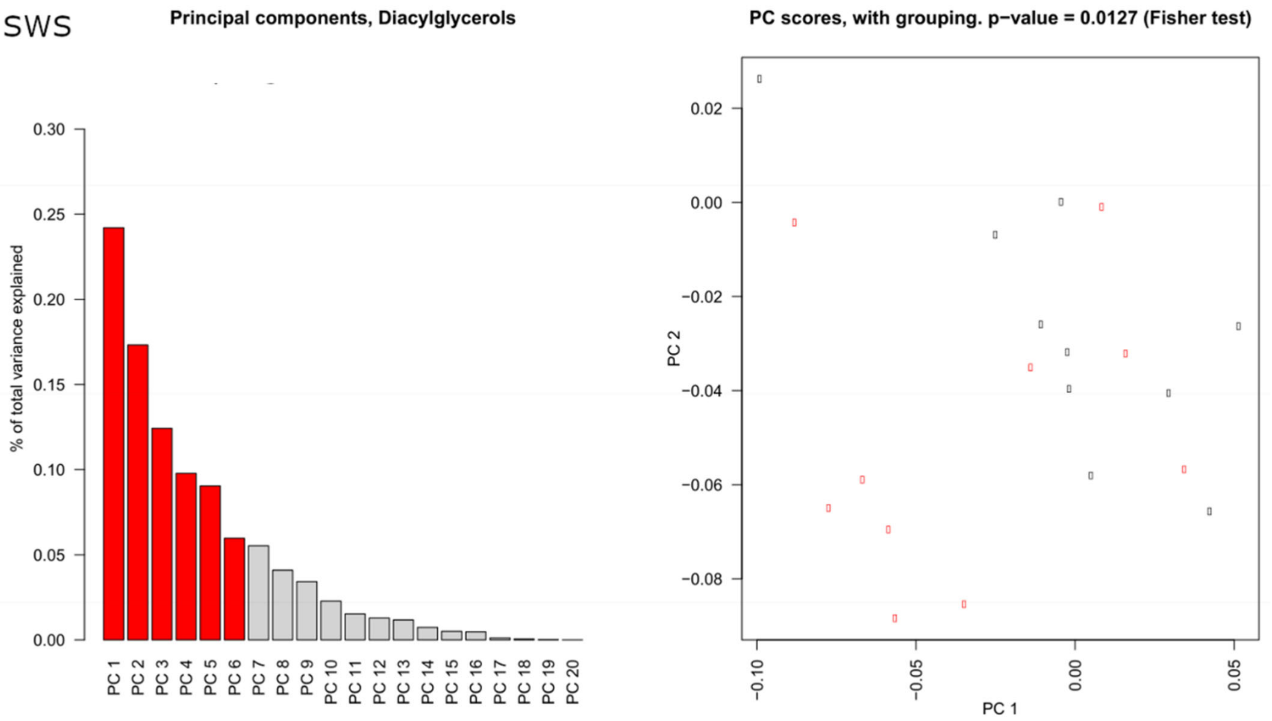

Supplementary Figure S13. Principal component analysis results on diacylglycerol in stimulated whole saliva. In the left panel, bar plot of the percentage of the total data variation explained by each estimated principal component. In the right panel, scatterplot of the principal component scores for primary Sjögren's syndrome (•) and control subjects (•). PC: principal component. Principal components analysis showed a significant difference of p=0.0127.

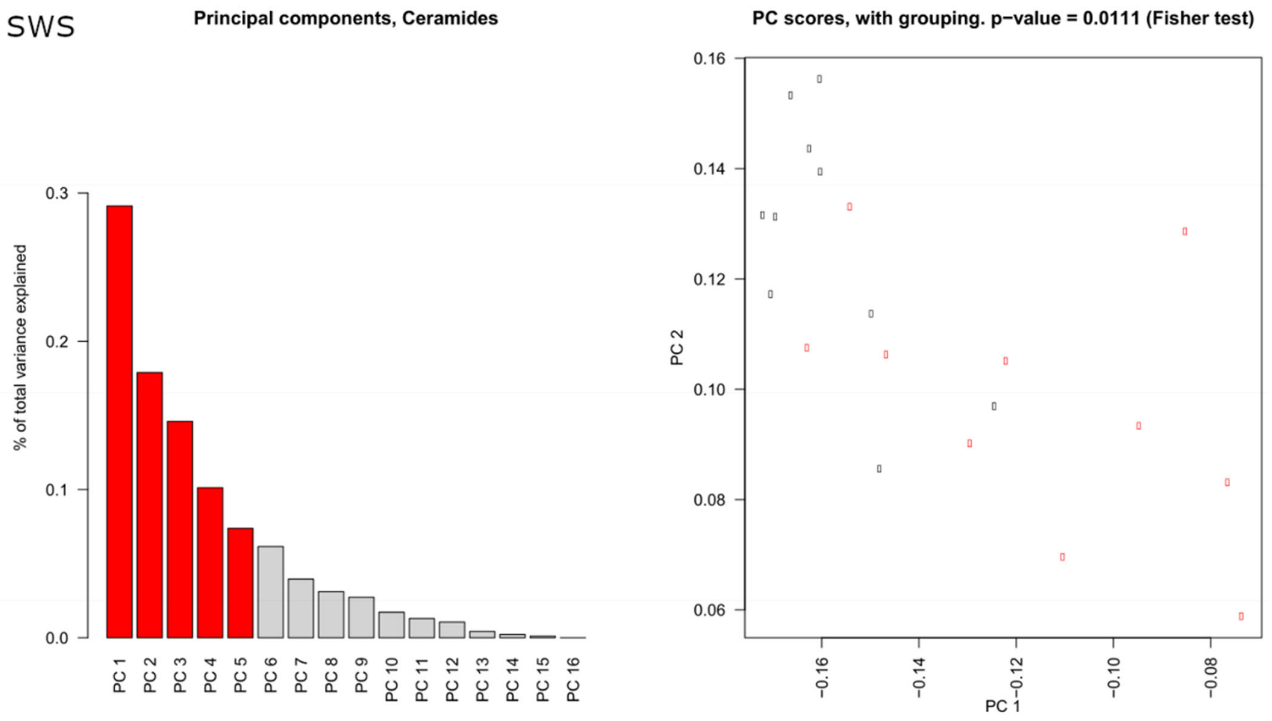

Supplementary Figure S14. Principal component analysis results on ceramide in stimulated whole saliva. In the left panel, bar plot of the percentage of the total data variation explained by each estimated principal component. In the right panel, scatterplot of the principal component scores for primary Sjögren's syndrome (•) and control subjects (•). PC: principal component. Principal components analysis showed a significant difference of p=0.0111.

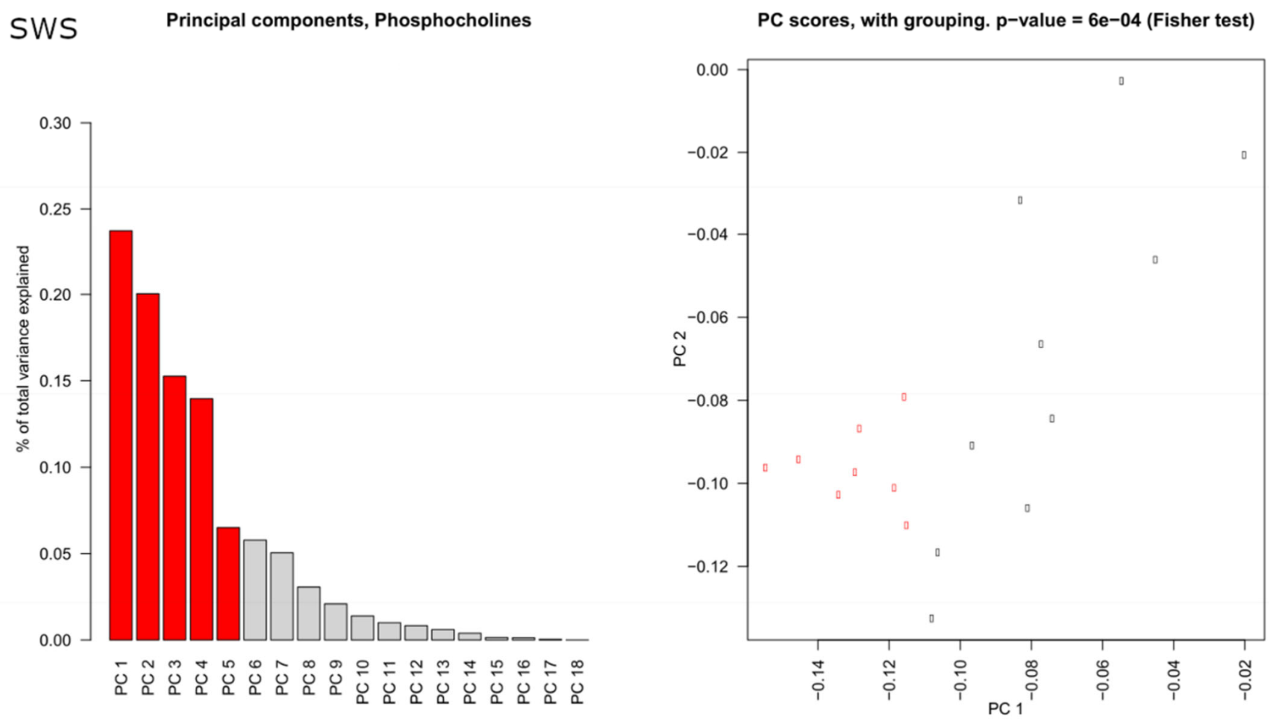

Supplementary Figure S15. Principal component analysis results on phosphocholine in stimulated whole saliva. In the left panel, bar plot of the percentage of the total data variation explained by each estimated principal component. In the right panel, scatterplot of the principal component scores for primary Sjögren's syndrome (•) and control subjects (•). PC: principal component. Principal components analysis showed a significant difference of p<0.01.

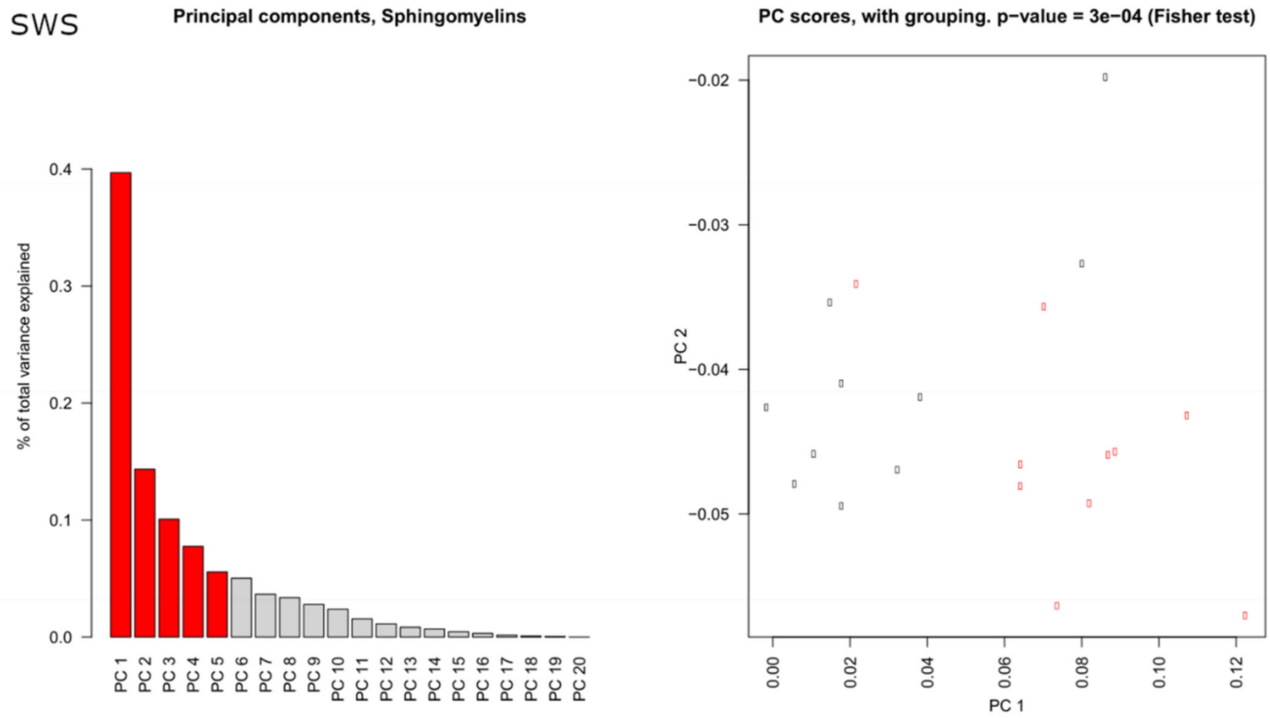

Supplementary Figure S16. Principal component analysis results on sphingomyelin in stimulated whole saliva. In the left panel, bar plot of the percentage of the total data variation explained by each estimated principal component. In the right panel, scatterplot of the principal component scores for primary Sjögren's syndrome (•) and control subjects (•). PC: principal component. Principal components analysis showed a significant difference of p<0.01.

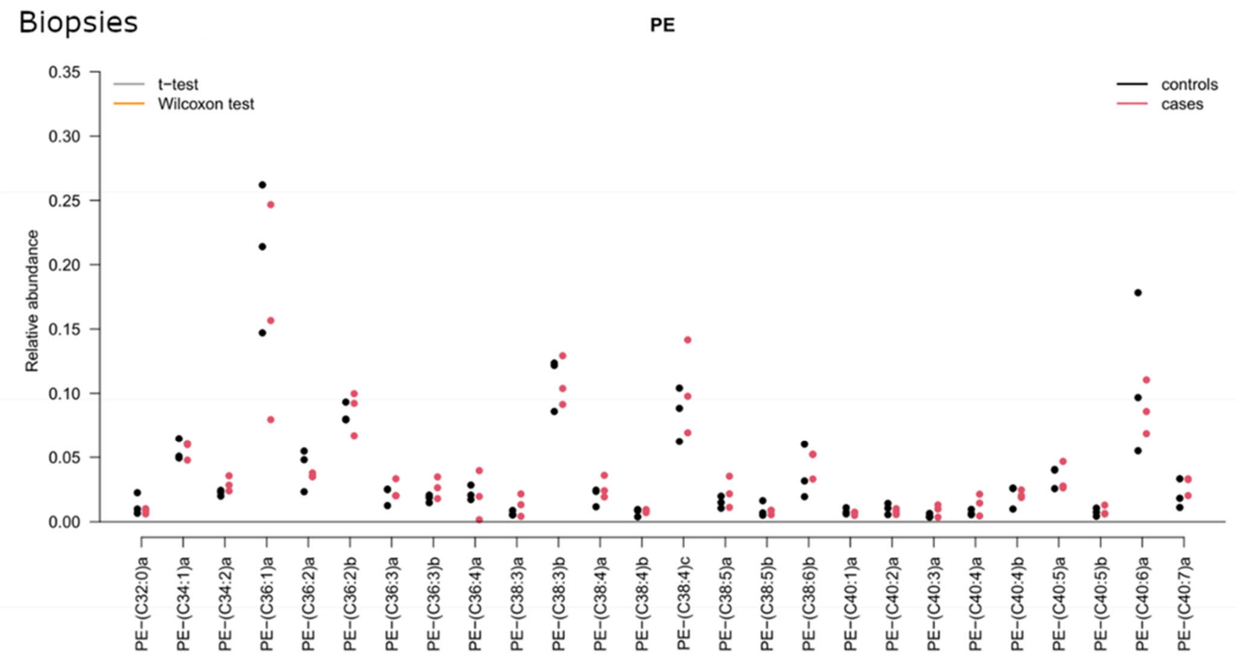

Supplementary Figure S17. Distribution of phosphatidylethanolamine in biopsies. PE: phosphatidylethanolamine. *p<0.05; **p<0.01.

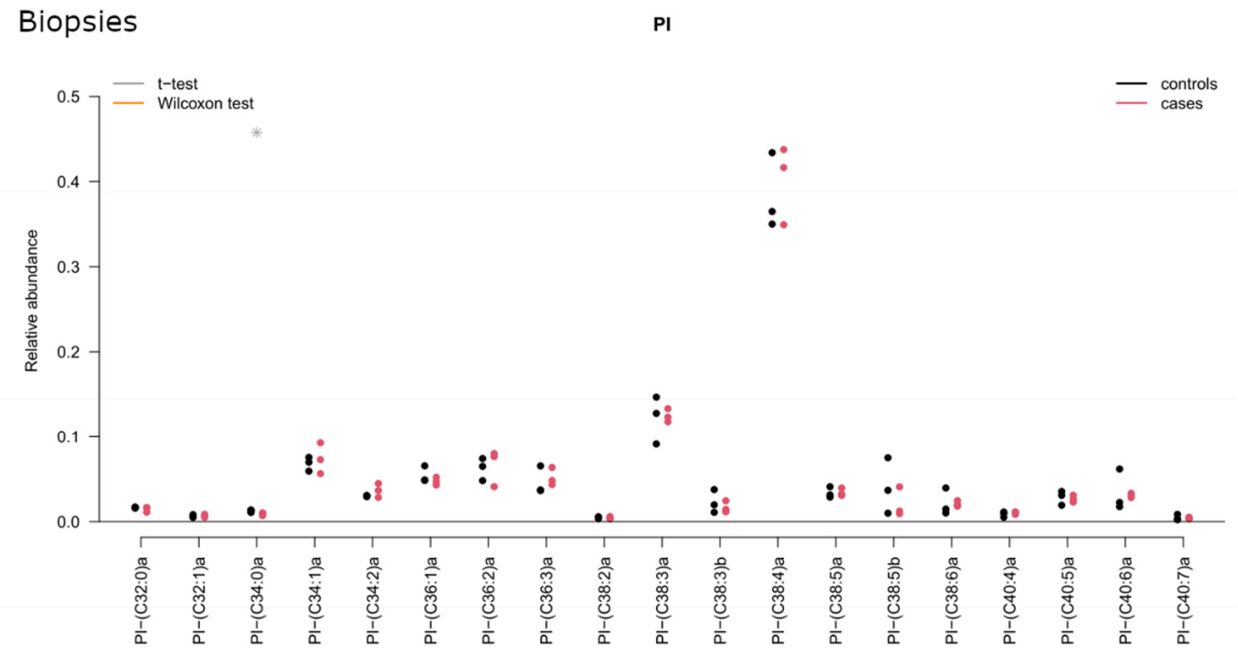

Supplementary Figure S18. Distribution of phosphatidylinositol in biopsies. PI: phosphatidylinositol. *p<0.05; **p<0.01.

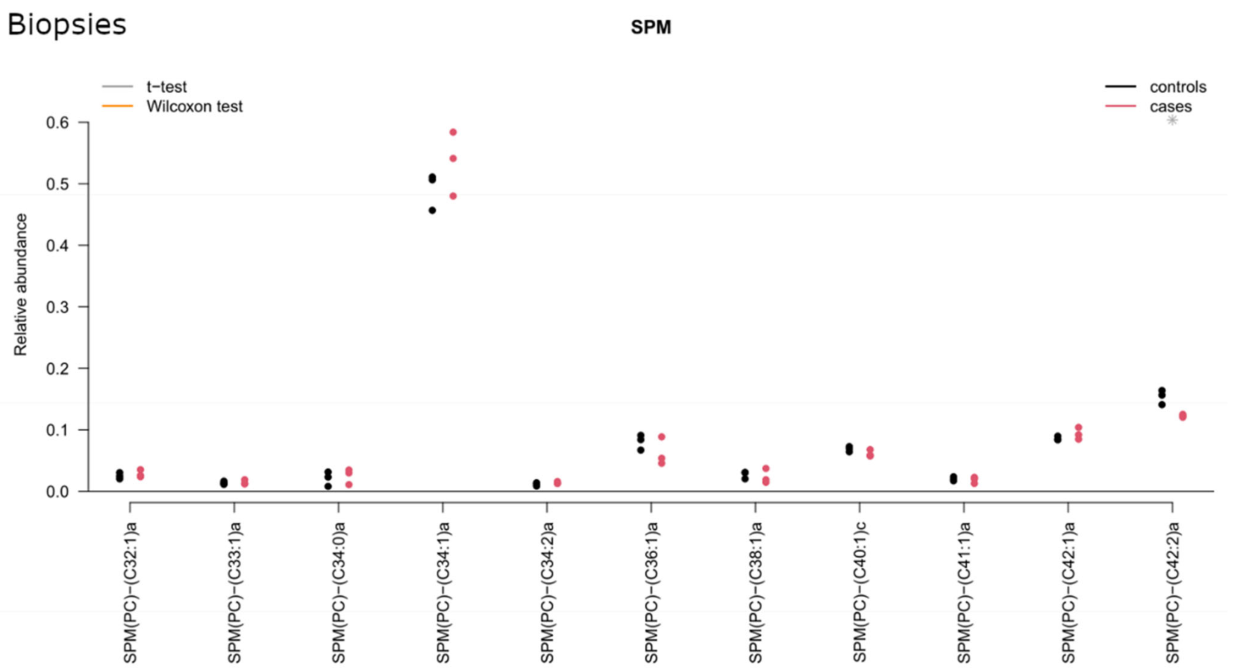

Supplementary Figure S19. Distribution of sphingomyelin in biopsies. SPM: sphingomyelin. *p<0.05; **p<0.01.

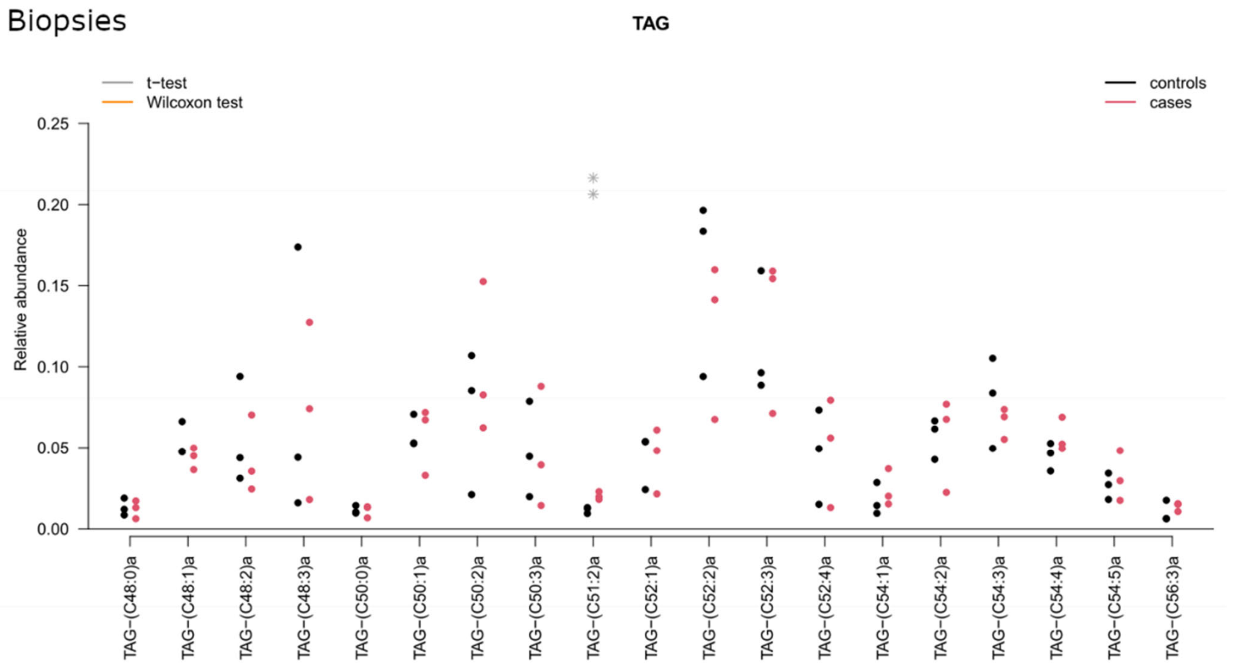

Supplementary Figure S20. Distribution of triacylglycerol in biopsies. TAG: triacylglycerol. *p<0.05; **p<0.01.

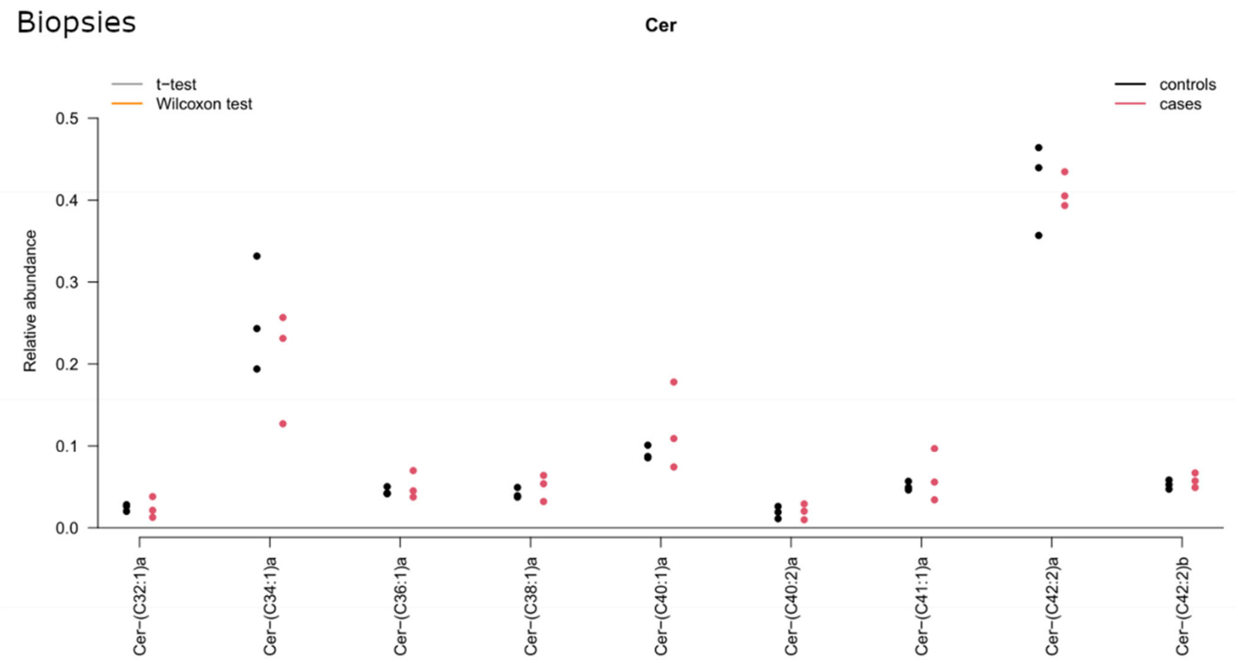

Supplementary Figure S21. Distribution of ceramide in biopsies. Cer: ceramide. *p<0.05; **p<0.01.

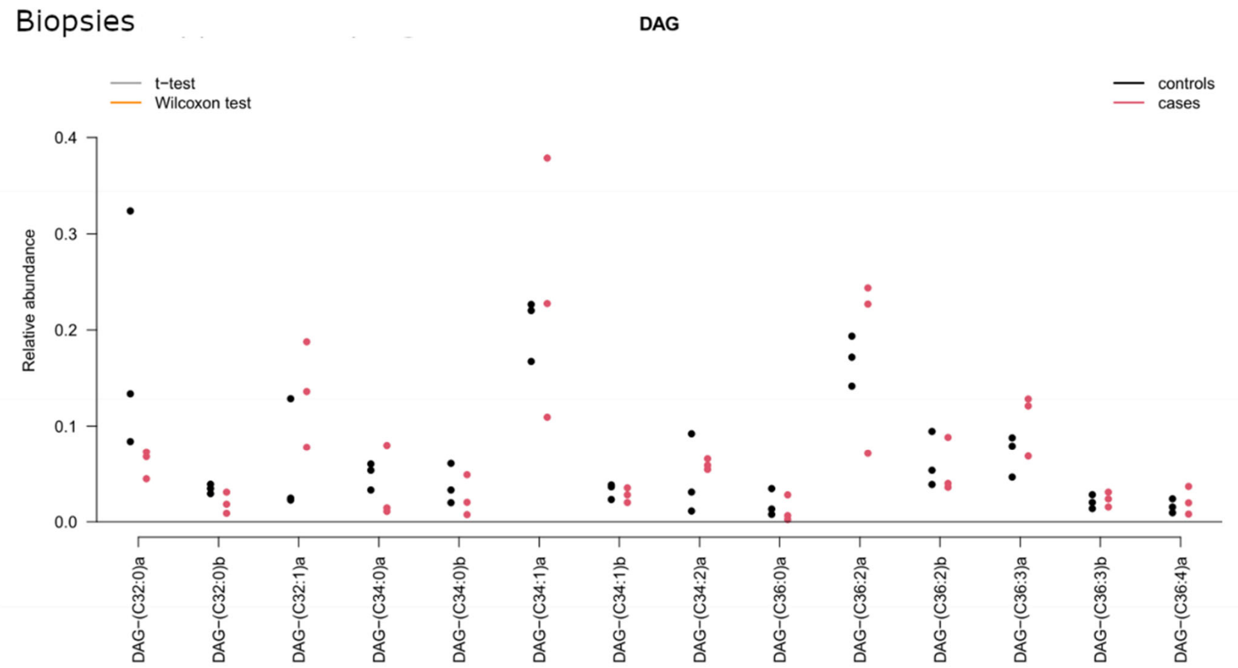

Supplementary Figure S22. Distribution of diacylglycerol in biopsies. DAG: diacylglycerol. *p<0.05; **p<0.01.

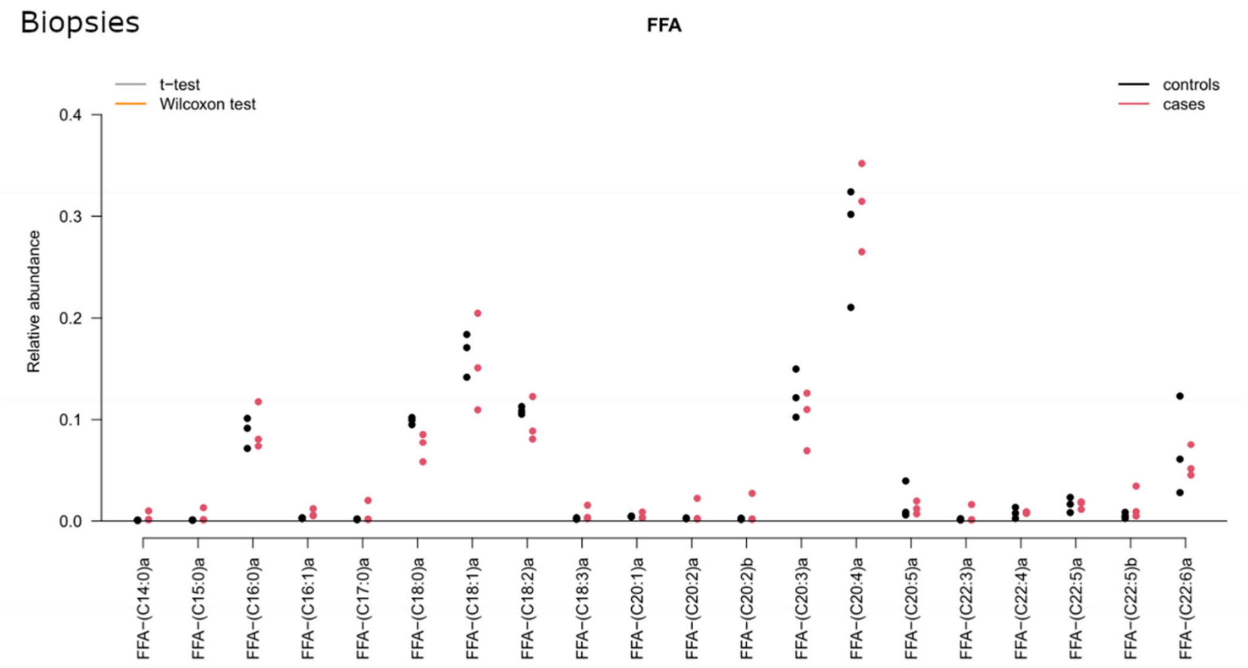

Supplementary Figure S23. Distribution of free fatty acids in biopsies. FFA: free fatty acid. *p<0.05; **p<0.01.

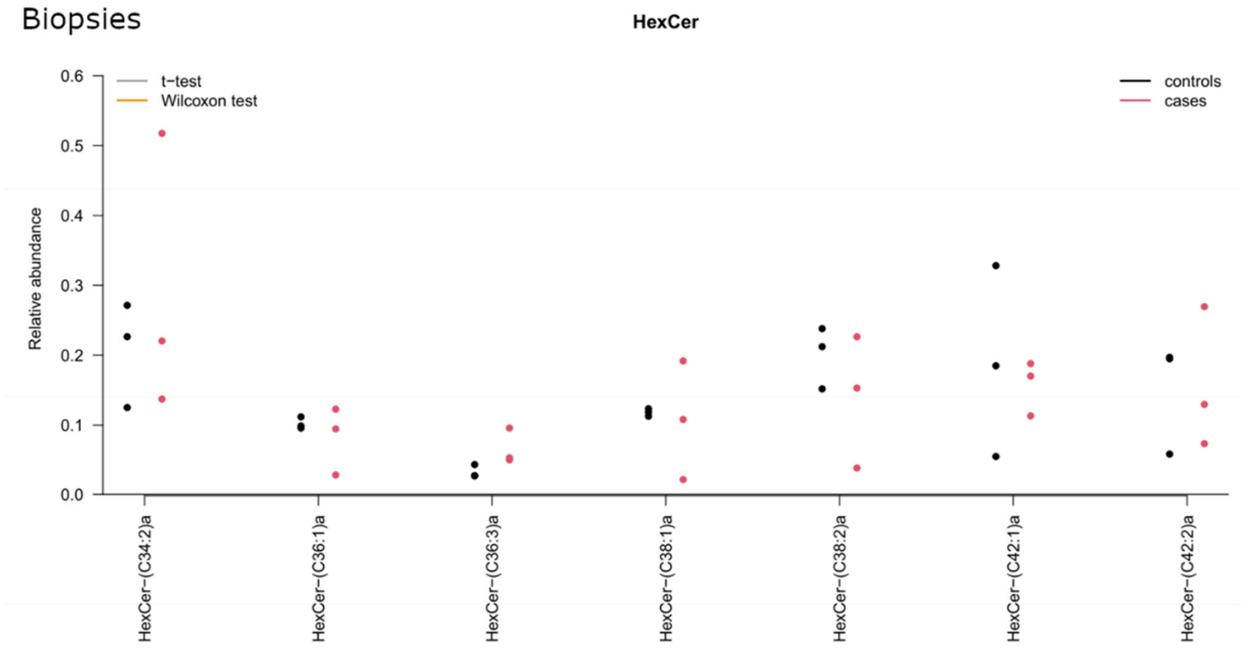

Supplementary Figure S24. Distribution of hexosylceramide in biopsies. HexCer: hexosylceramide. *p<0.05; **p<0.01.

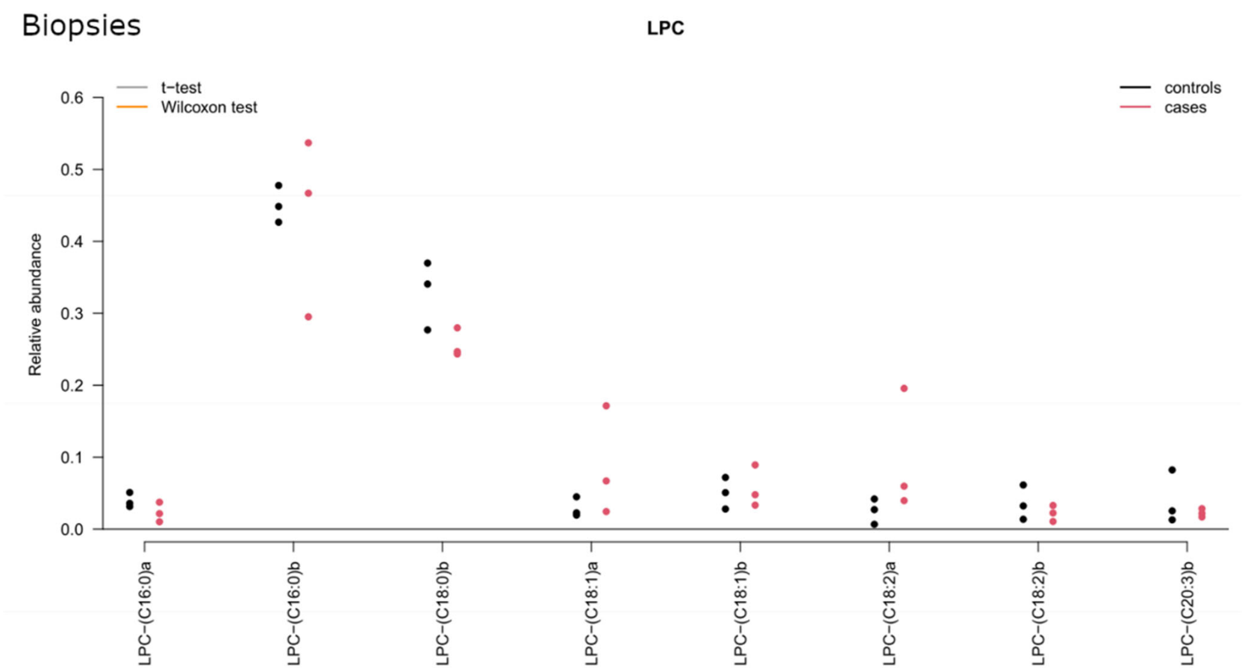

Supplementary Figure S25. Distribution of lysophosphatidylcholine in biopsies. LPC: lysophosphatidylcholine. *p<0.05; **p<0.01.

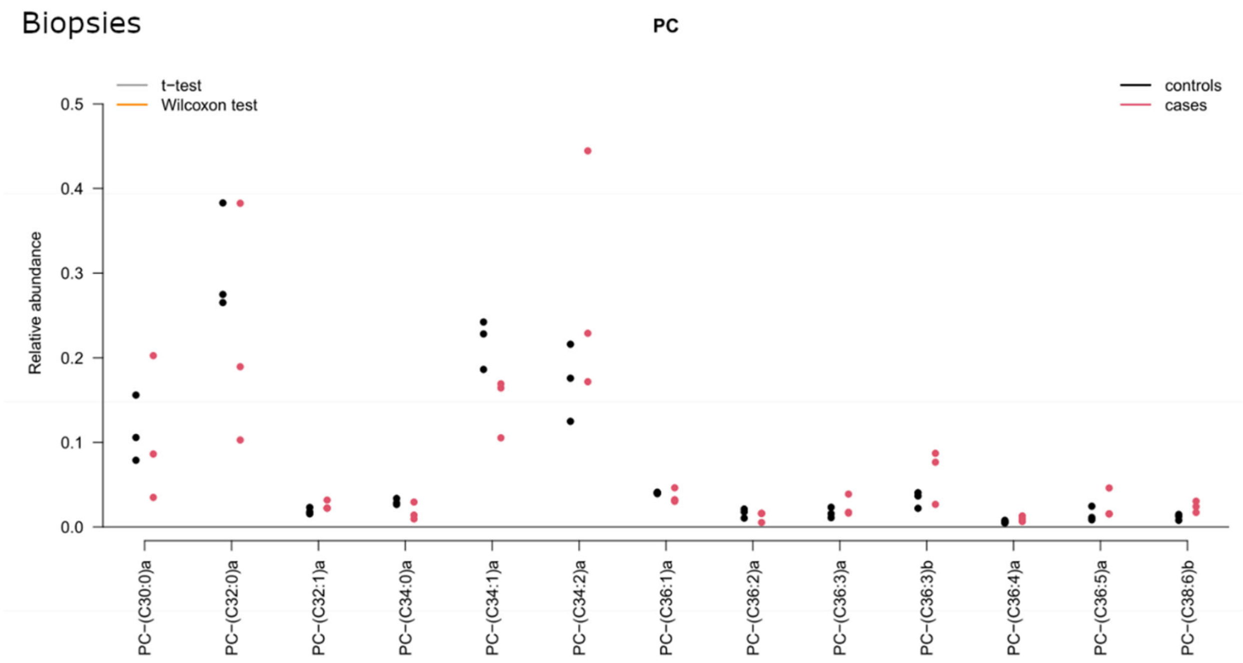

Supplementary Figure S26. Distribution of phosphatidylcholine in biopsies. PC: phosphatidylcholine. *p<0.05; **p<0.01.

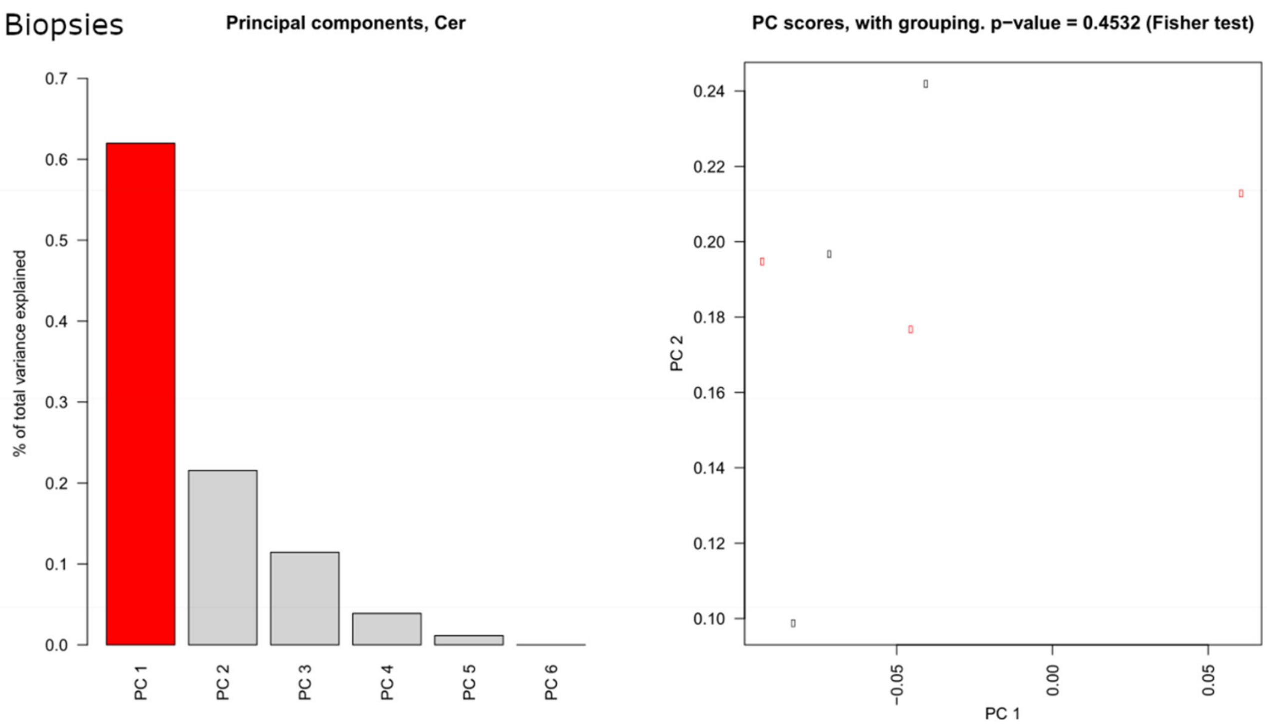

Supplementary Figure S27. Principal component analysis results on ceramide in biopsies. In the left panel, bar plot of the percentage of the total data variation explained by each estimated principal component. In the right panel, scatterplot of the principal component scores for pSS (•) and control subjects (•). Principal components analysis showed an insignificant difference of p=0.45. Cer: ceramide. PC: principal component.

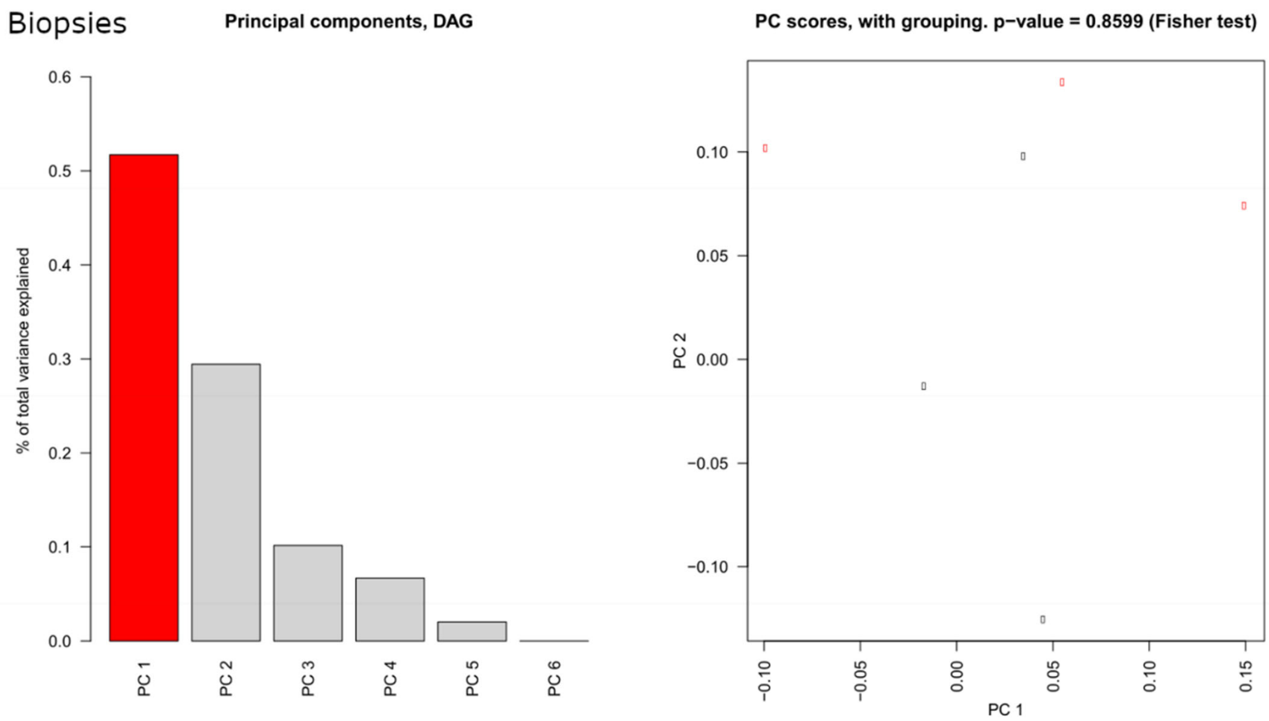

Supplementary Figure S28. Principal component analysis results on diacylglycerol in biopsies. In the left panel, bar plot of the percentage of the total data variation explained by each estimated principal component. In the right panel, scatter-plot of the principal component scores for pSS (•) and control subjects (•). Principal components analysis showed an insignificant difference of p=0.86. DAG: diacylglycerol. PC: principal component.

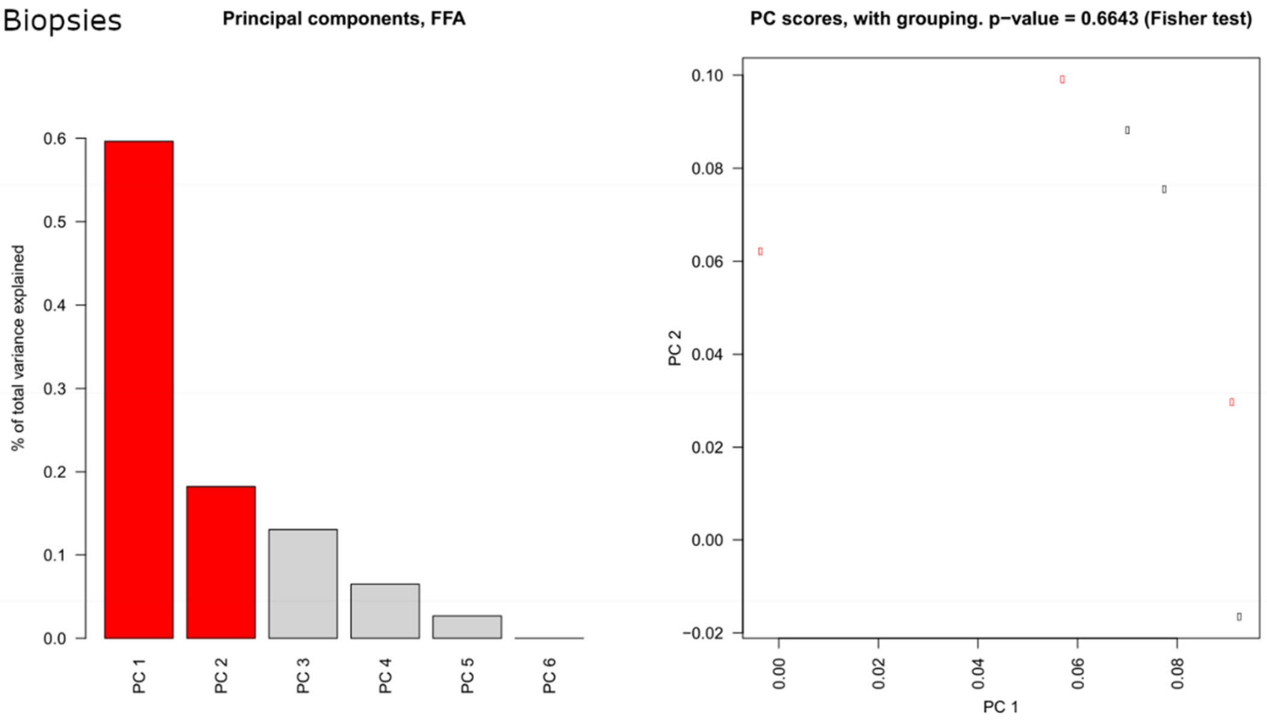

Supplementary Figure S29. Principal component analysis results on free fatty acid in biopsies. In the left panel, bar plot of the percentage of the total data variation explained by each estimated principal component. In the right panel, scatterplot of the principal component scores for pSS (•) and control subjects (•). Principal components analysis showed an insignificant difference of p=0.66. FFA: free fatty acid. PC: principal component.

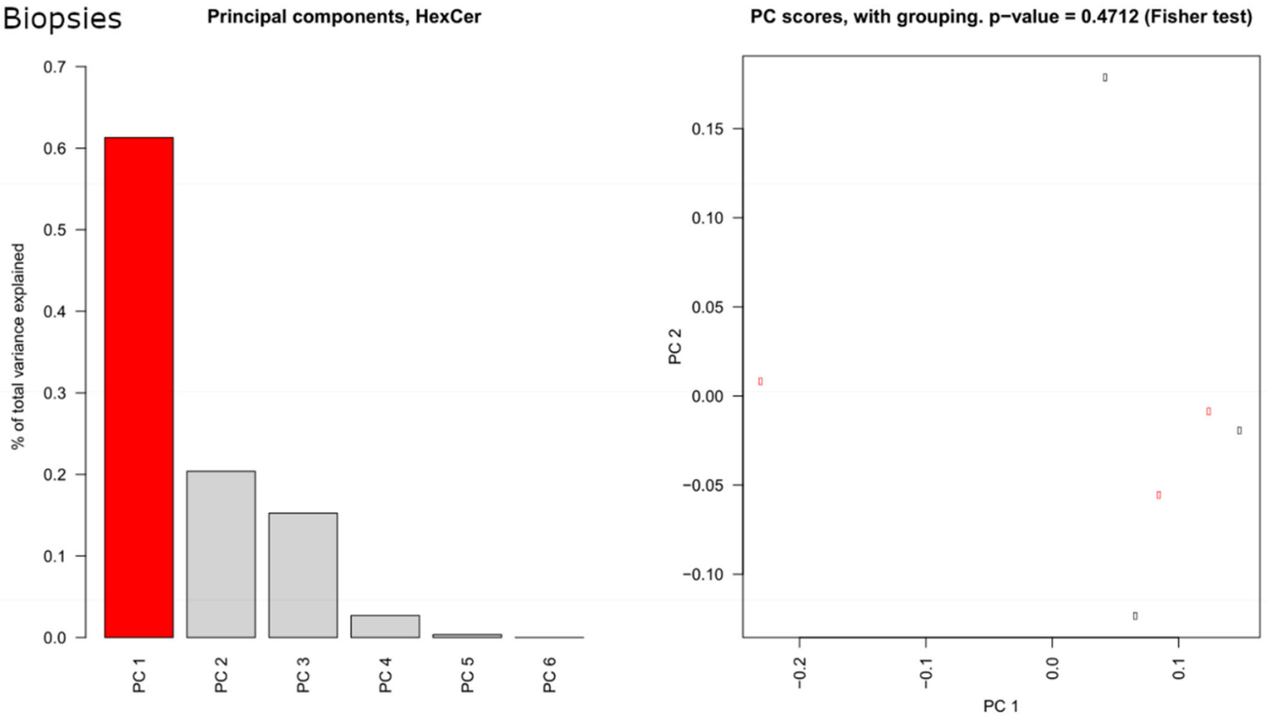

Supplementary Figure S30. Principal component analysis results on hexosylceramide in biopsies. In the left panel, bar plot of the percentage of the total data variation explained by each estimated principal component. In the right panel, scatterplot of the principal component scores for pSS (•) and control subjects (•). Principal components analysis showed an insignificant difference of p=0.47. HexCer: hexosylceramide. PC: principal component.

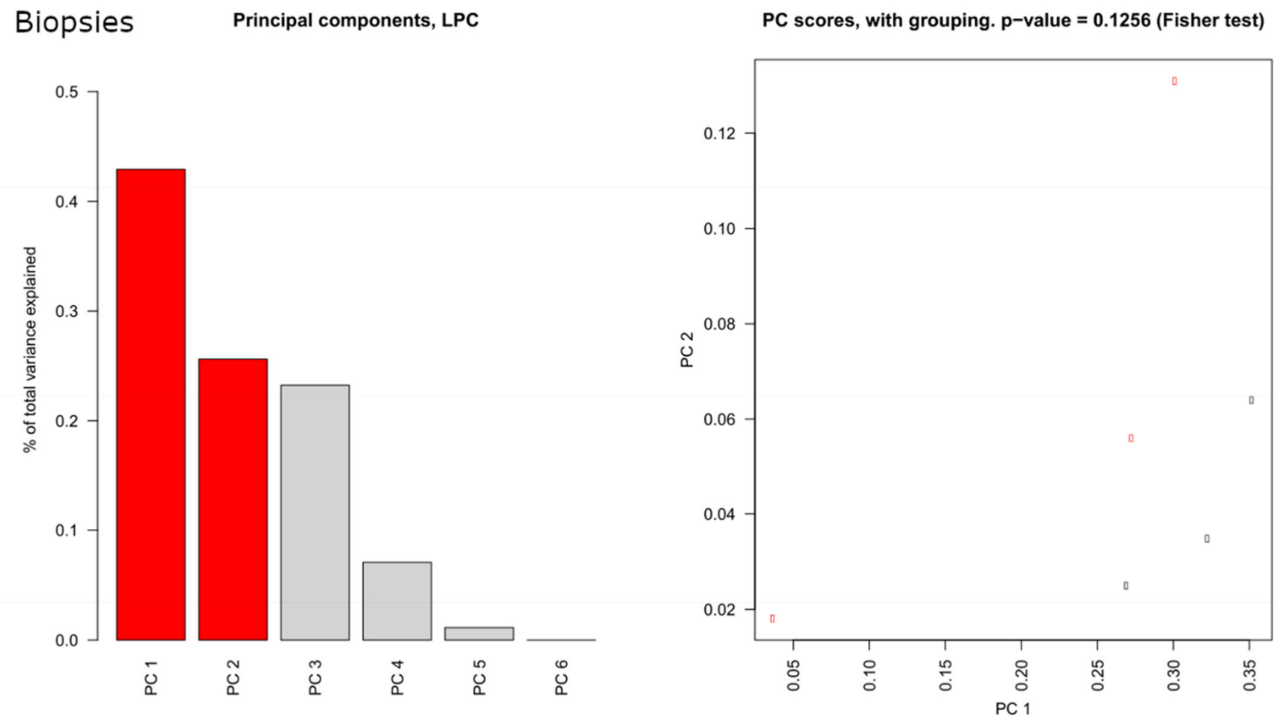

Supplementary Figure S31. Principal component analysis results on lysophosphatidylcholine in biopsies. In the left panel, bar plot of the percentage of the total data variation explained by each estimated principal component. In the right panel, scatterplot of the principal component scores for pSS (•) and control subjects (•). Principal components analysis showed an insignificant difference of p=0.13. LPC: lysophosphatidylcholine. PC: principal component.

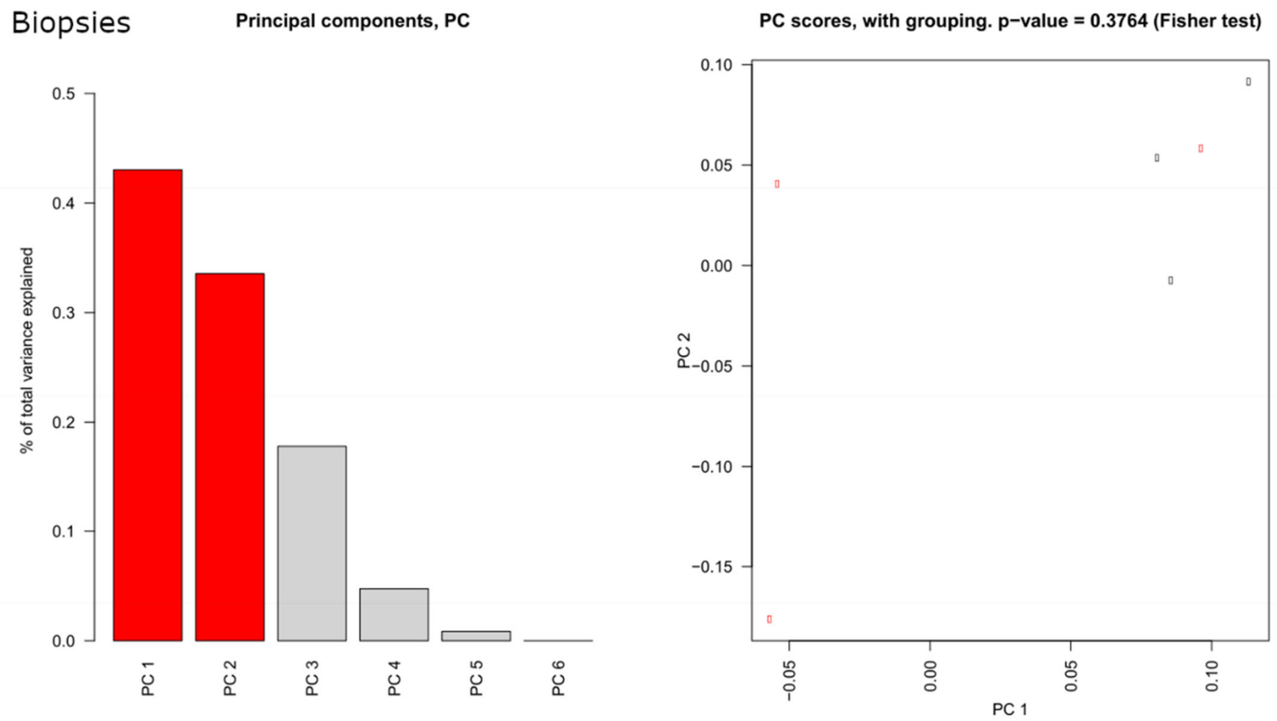

Supplementary Figure S32. Principal component analysis results on phosphatidylcholine in biopsies. In the left panel, bar plot of the percentage of the total data variation explained by each estimated principal component. In the right panel, scatterplot of the principal component scores for pSS (•) and control subjects (•). Principal components analysis showed an insignificant difference of p=0.38. PC: phosphatidylcholine. PC 1-6: principal component 1-6.

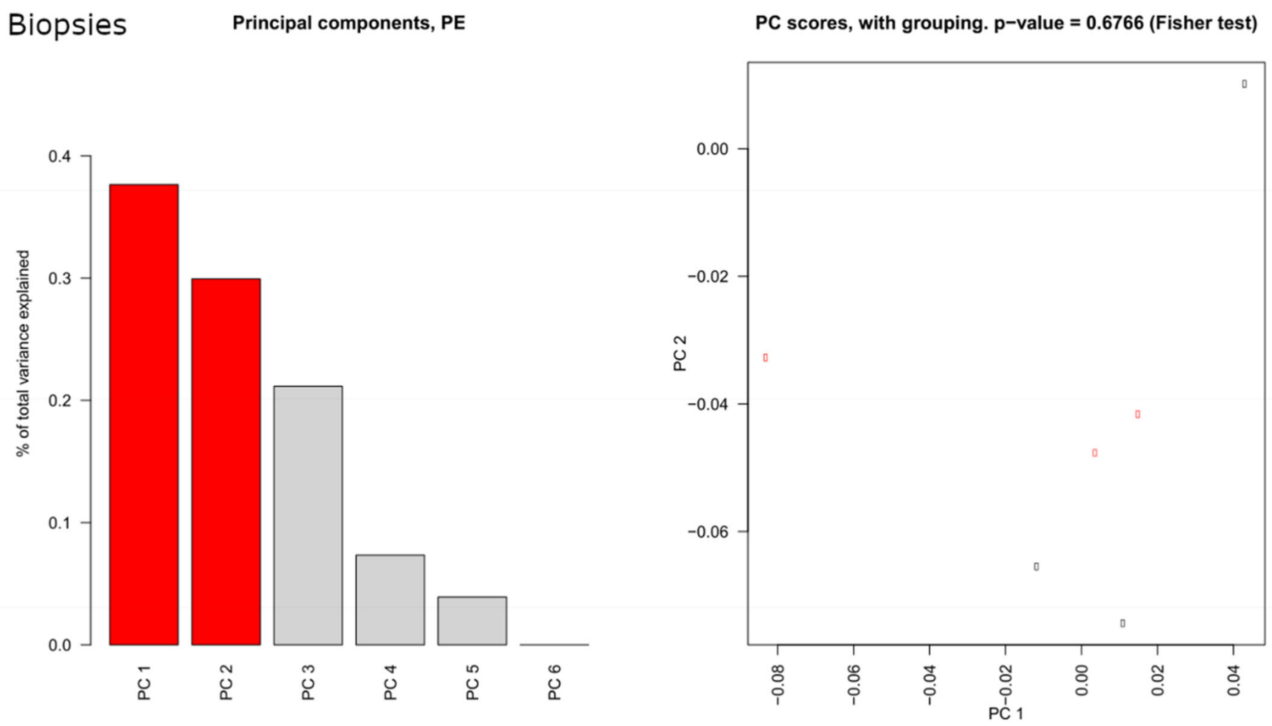

Supplementary Figure S33. Principal component analysis results on phosphatidylethanolamine in biopsies. In the left panel, bar plot of the percentage of the total data variation explained by each estimated principal component. In the right panel, scatterplot of the principal component scores for pSS (•) and control subjects (•). Principal components analysis showed an insignificant difference of p=0.68. PE: phosphatidylethanolamine. PC: principal component.

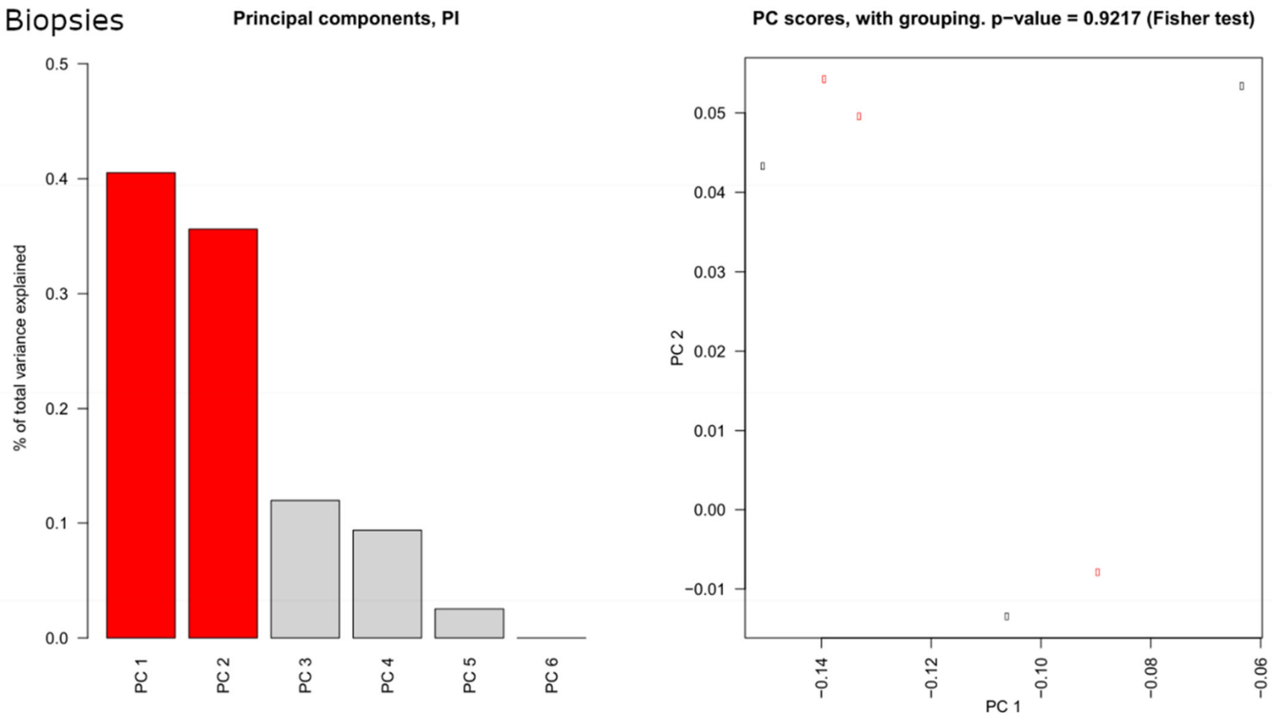

Supplementary Figure S34. Principal component analysis results on phosphatidylinositol in biopsies. In the left panel, bar plot of the percentage of the total data variation explained by each estimated principal component. In the right panel, scatterplot of the principal component scores for pSS (•) and control subjects (•). Principal components analysis showed an insignificant difference of p=0.92. PI: phosphatidylinositol. PC: principal component.

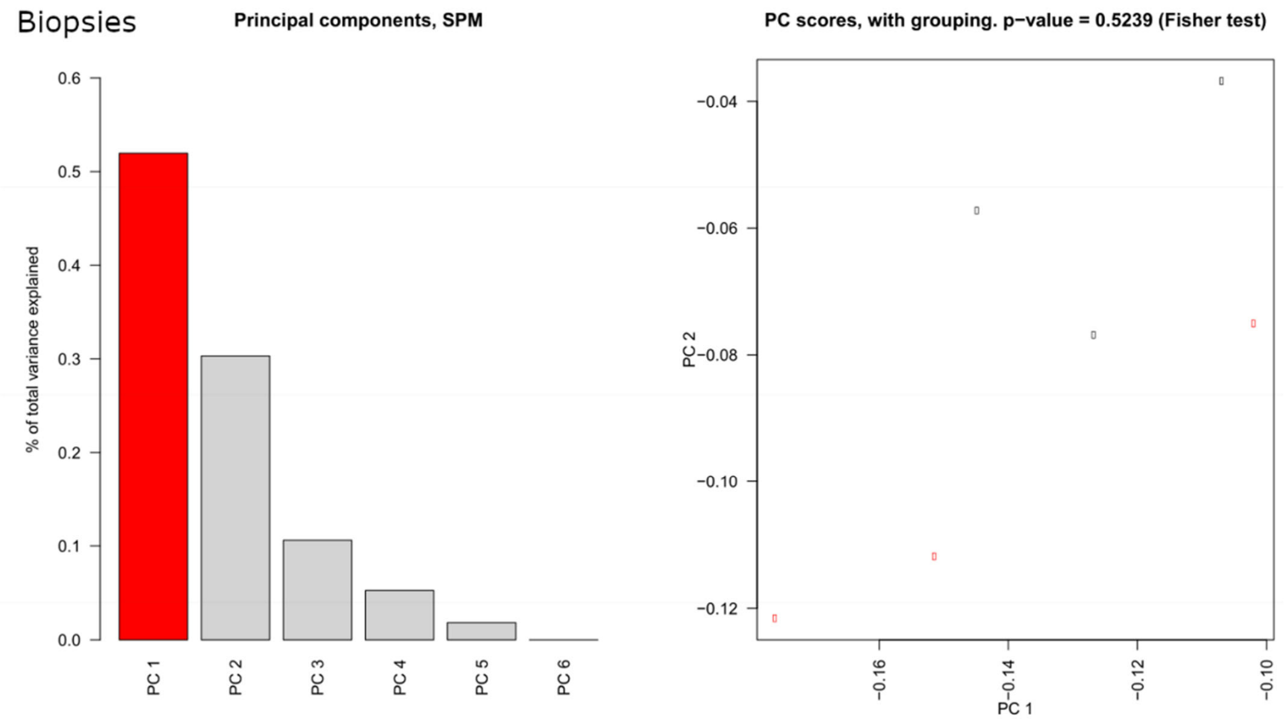

Supplementary Figure S35. Principal component analysis results on sphingomyelin in biopsies. In the left panel, bar plot of the percentage of the total data variation explained by each estimated principal component. In the right panel, scatter-plot of the principal component scores for pSS (•) and control subjects (•). Principal components analysis showed an insignificant difference of p=0.52. SPM: sphingomyelin. PC: principal component.

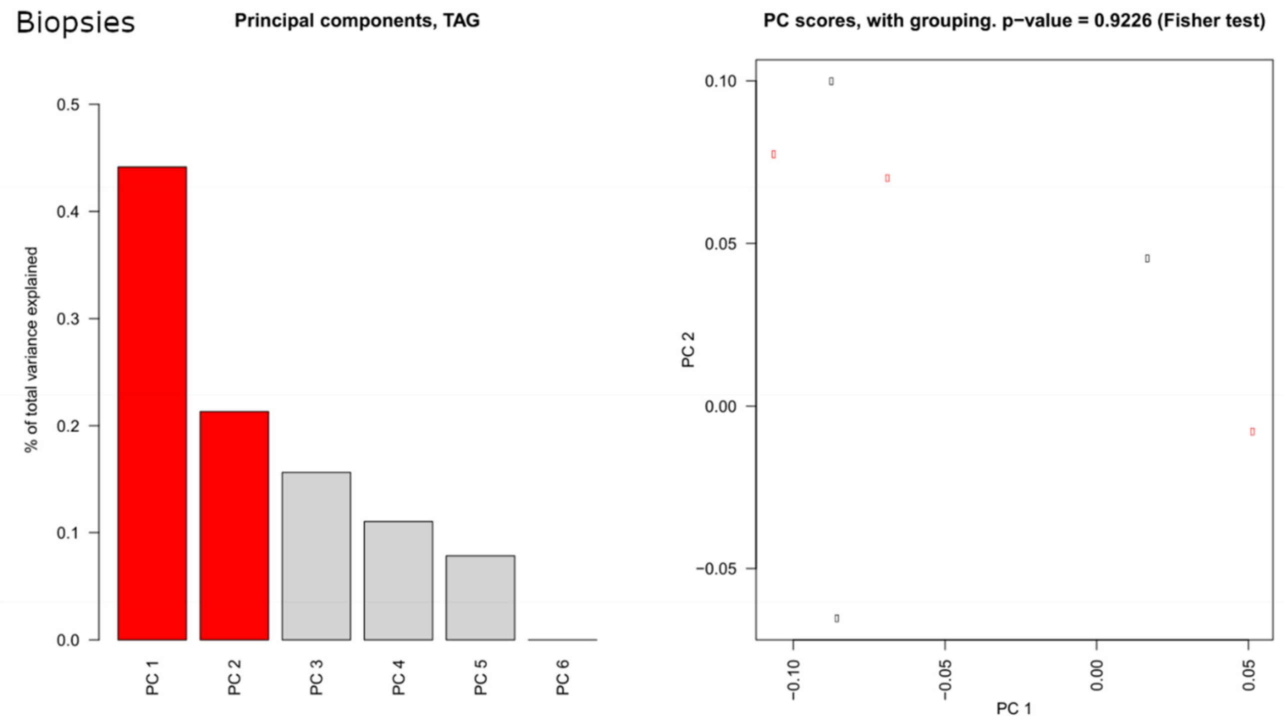

Supplementary Figure S36. Principal component analysis results on triacylglycerol in biopsies. In the left panel, bar plot of the percentage of the total data variation explained by each estimated principal component. In the right panel, scatter-plot of the principal component scores for pSS (•) and control subjects (•). Principal components analysis showed an insignificant difference of p=0.92. TAG: triacylglycerol. PC: principal component.

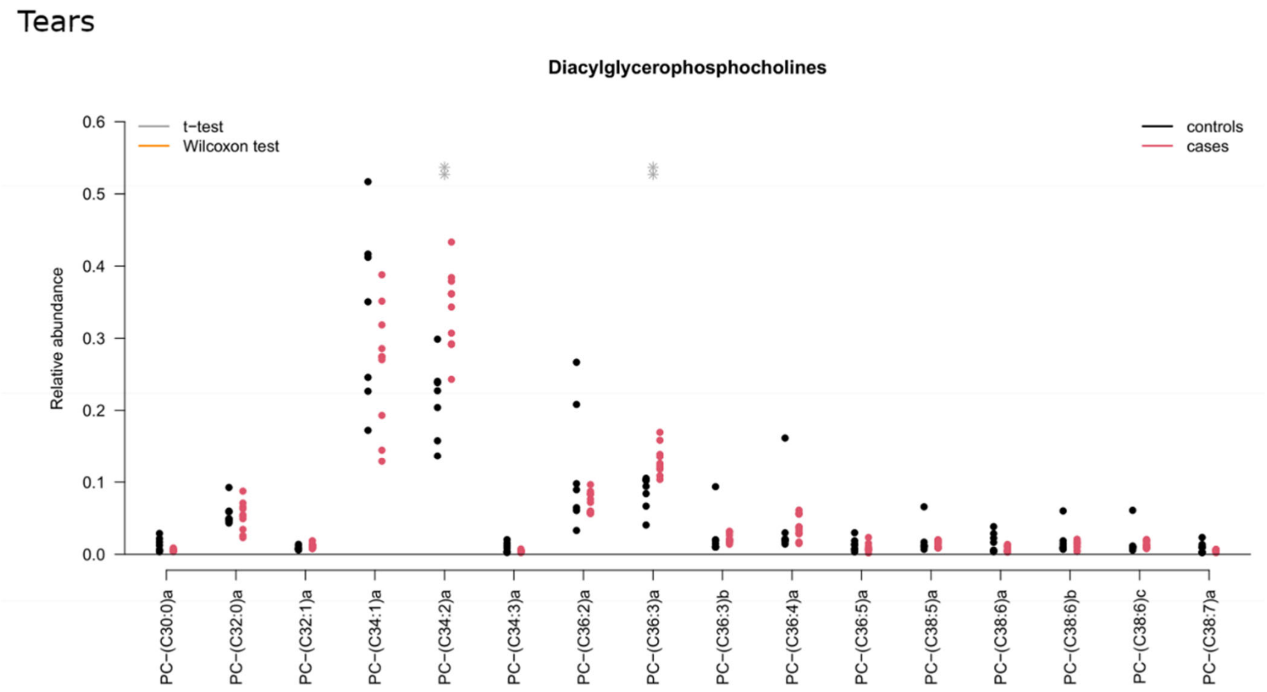

Scheme 37. Distribution of diacylglycerophosphocholine in tears. *p<0.05; **p<0.01.

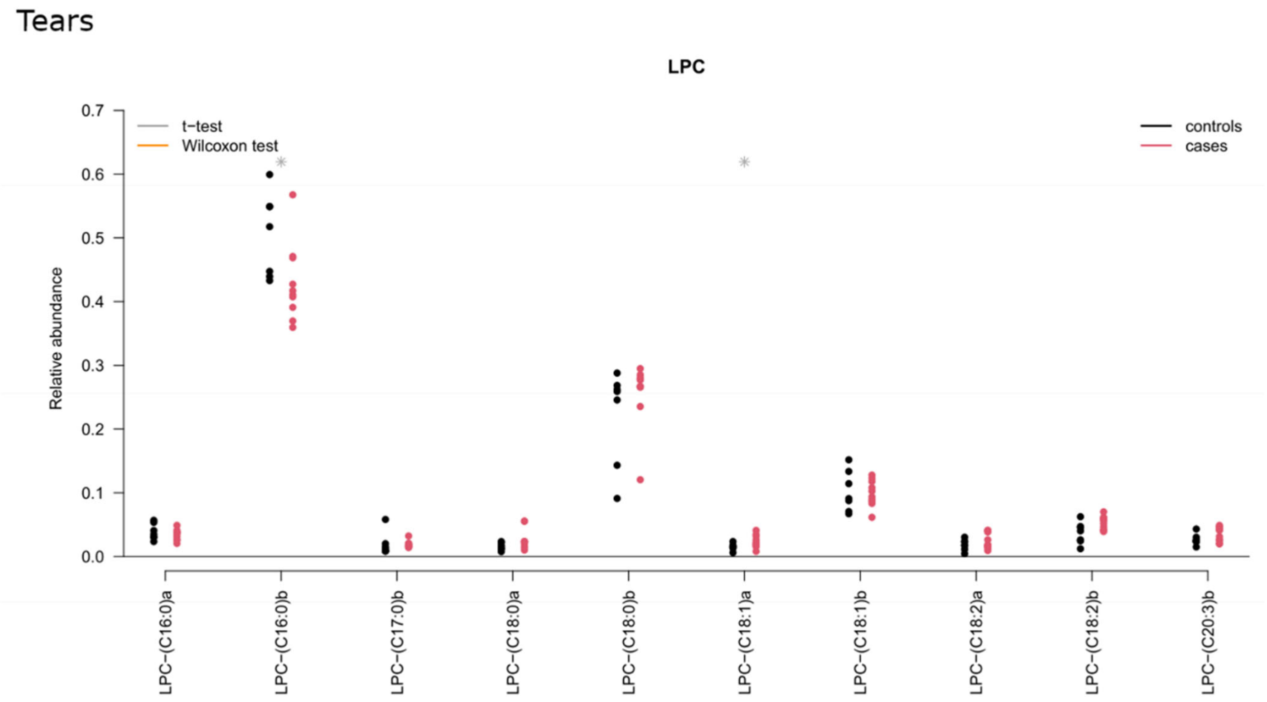

Supplementary Figure S38. Distribution of lysophosphatidylcholine in tears. LPC: lysophosphatidylcholine. *p<0.05; **p<0.01.

Tears

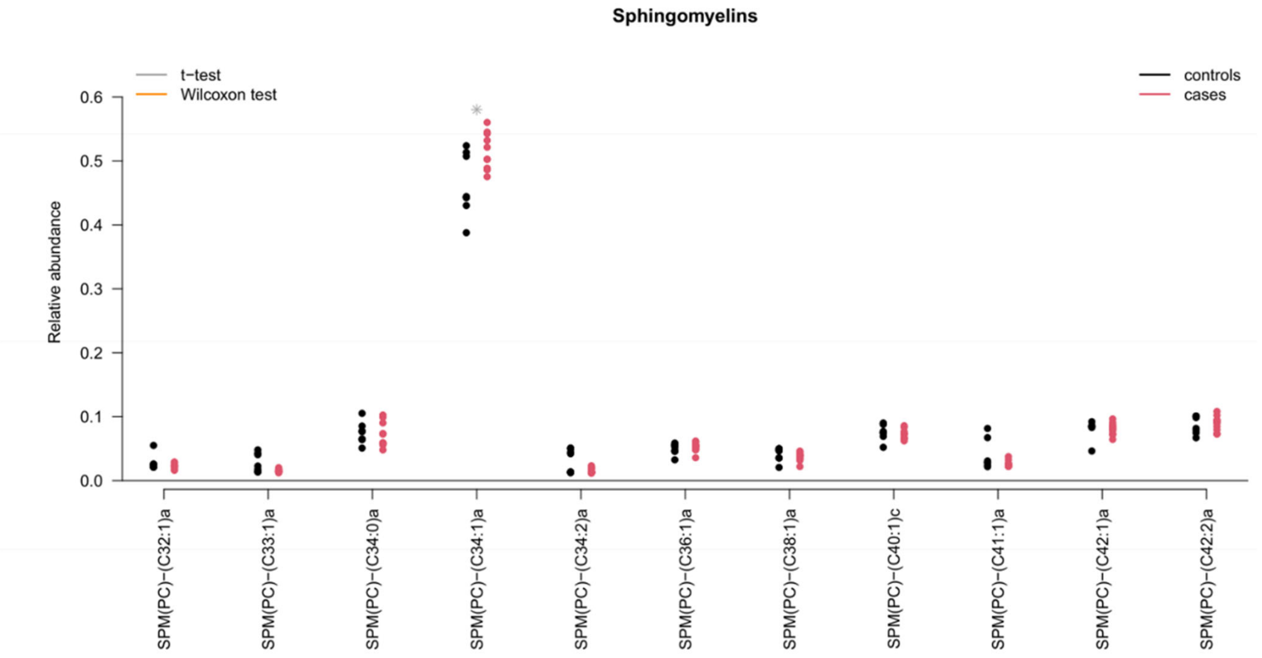

Supplementary Figure S39. Distribution of sphingomyelin in tears. SPM: sphingomyelin. *p<0.05; **p<0.01.

Tears

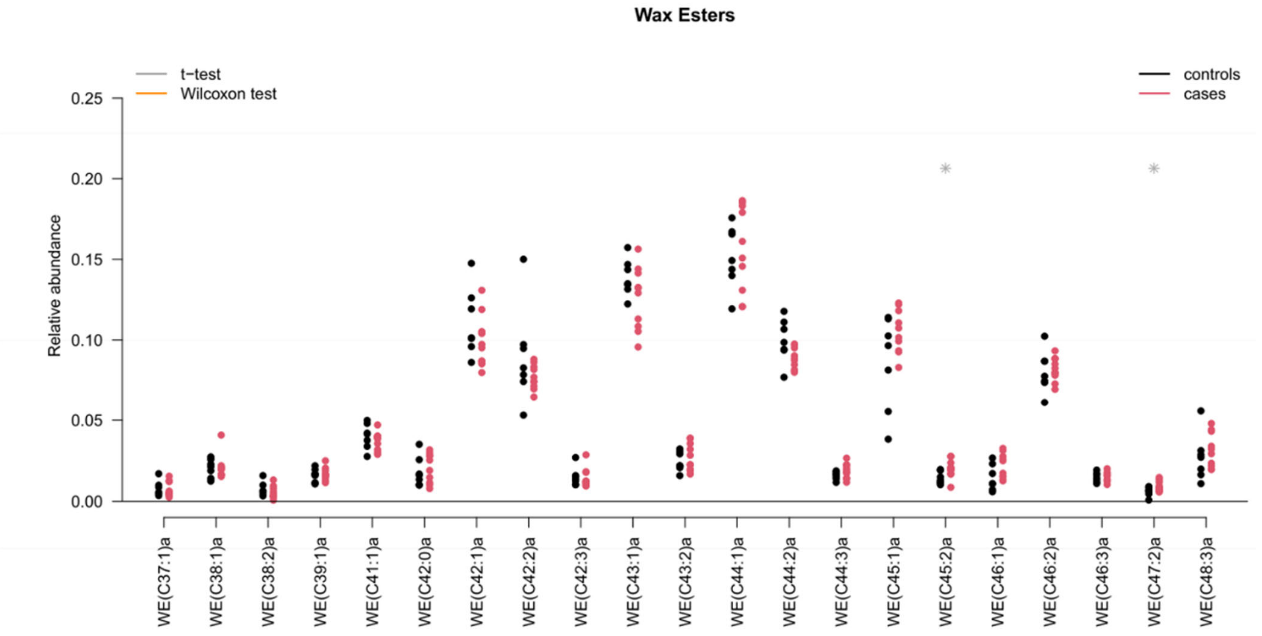

Supplementary Figure S40. Distribution of wax ester in tears. WE: wax ester. *p<0.05; **p<0.01.

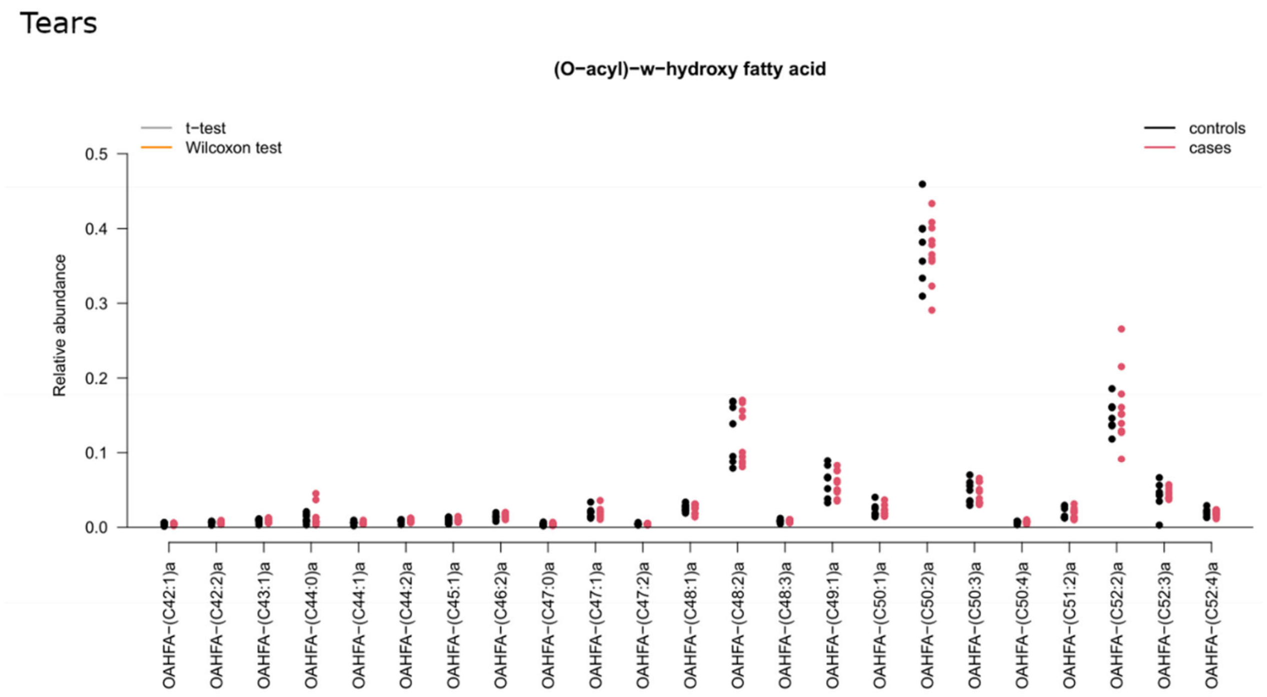

Supplementary Figure S41. Distribution of (O-acyl)-ω-hydroxy fatty acid in tears. OAHFA: (O-acyl)-ω-hydroxy fatty acid. *p<0.05; **p<0.01.

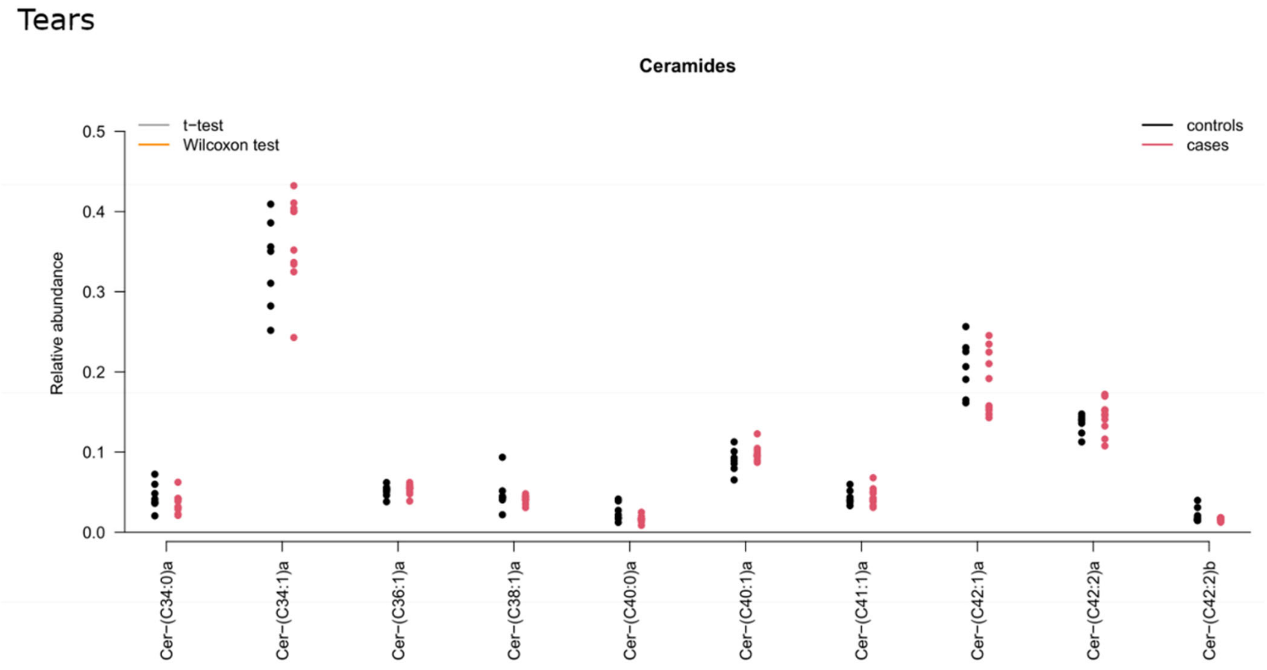

Supplementary Figure S42. Distribution of ceramide in tears. Cer: ceramide. *p<0.05; **p<0.01.

Tears

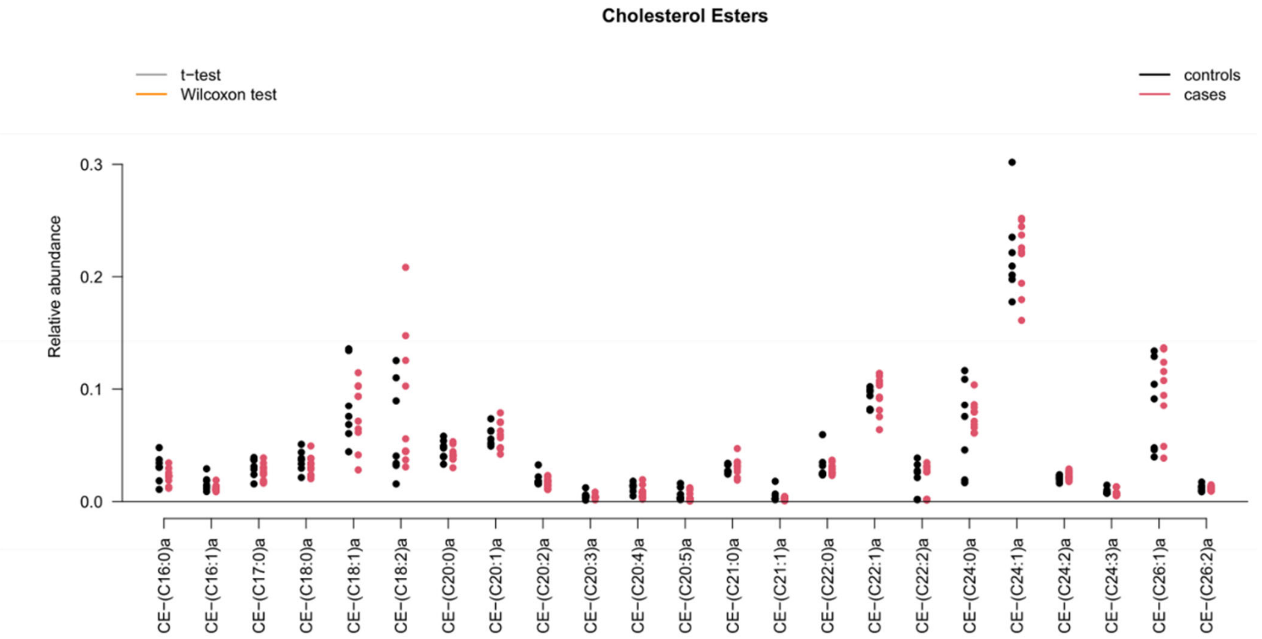

Supplementary Figure S43. Distribution of cholesterol ester in tears. CE: cholesterol ester. *p<0.05; **p<0.01.

Tears

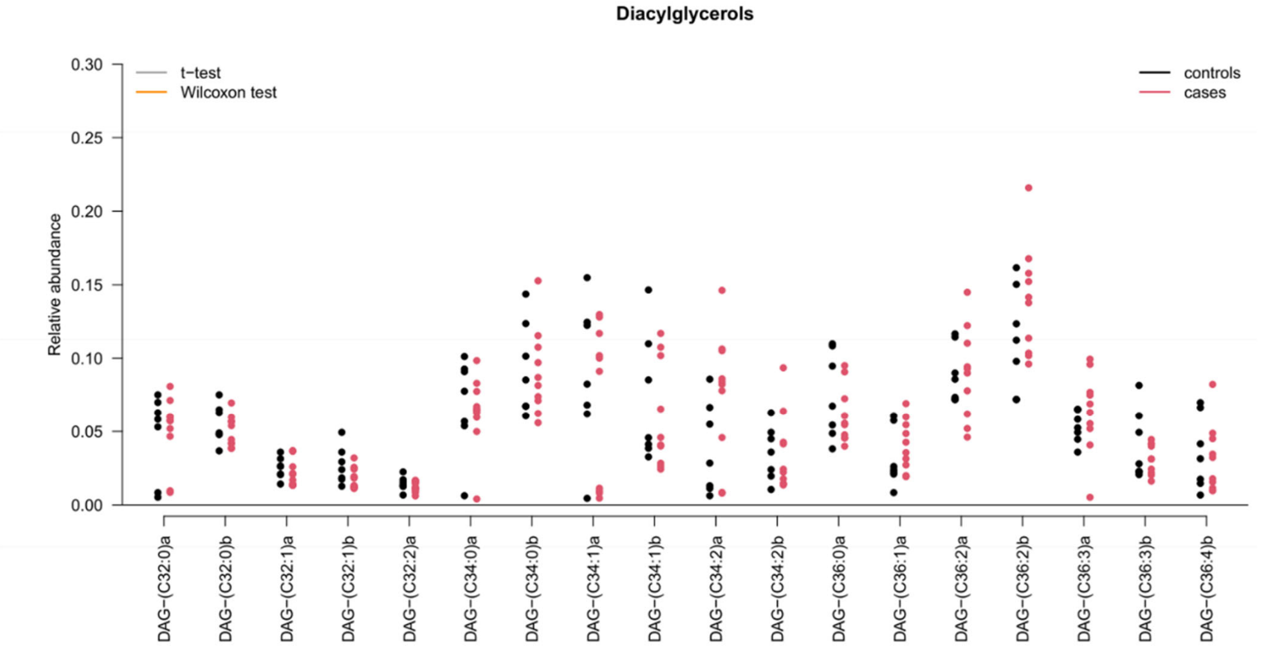

Supplementary Figure S44. Distribution of diacylglycerol in tears. DAG: diacylglycerol. *p<0.05; **p<0.01.

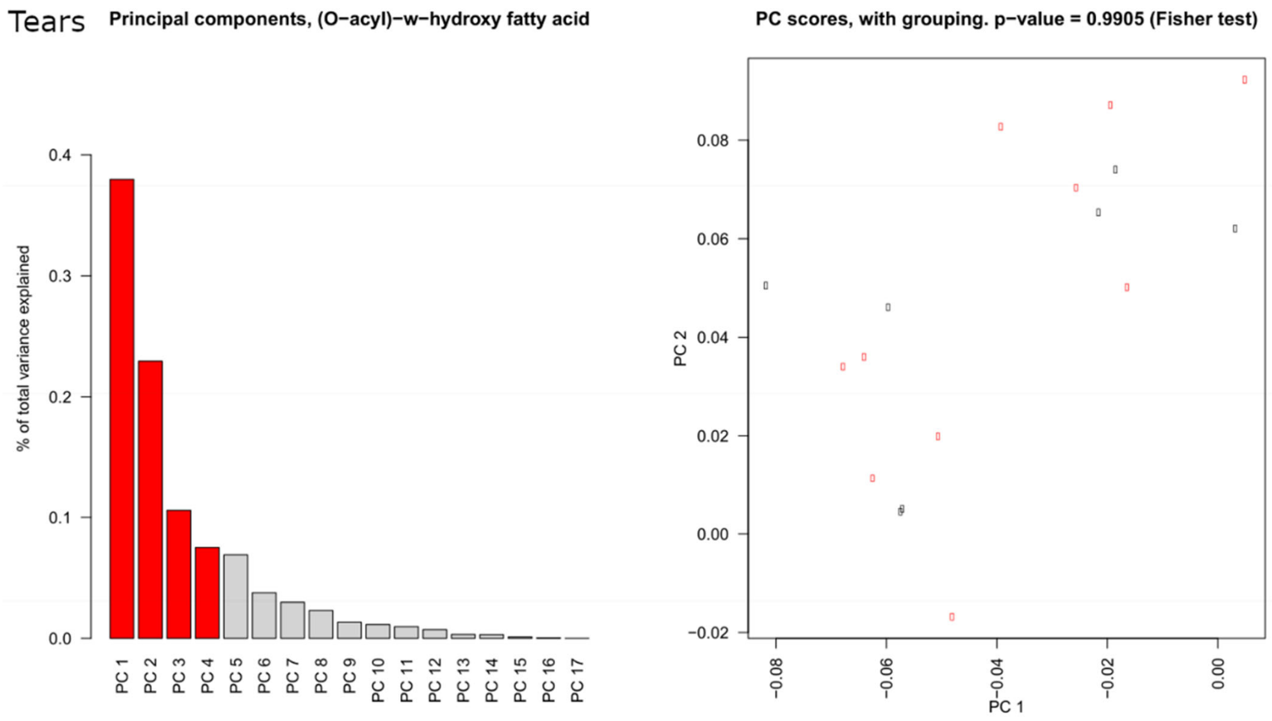

Supplementary Figure S45. Principal component analysis results on (O-acyl)-ω-hydroxy fatty acid in tears. In the left panel, bar plot of the percentage of the total data variation explained by each estimated principal component. In the right panel, scatterplot of the principal component scores for pSS (•) and control subjects (•). Principal components analysis showed an insignificant difference of p=0.99. PC: principal component.

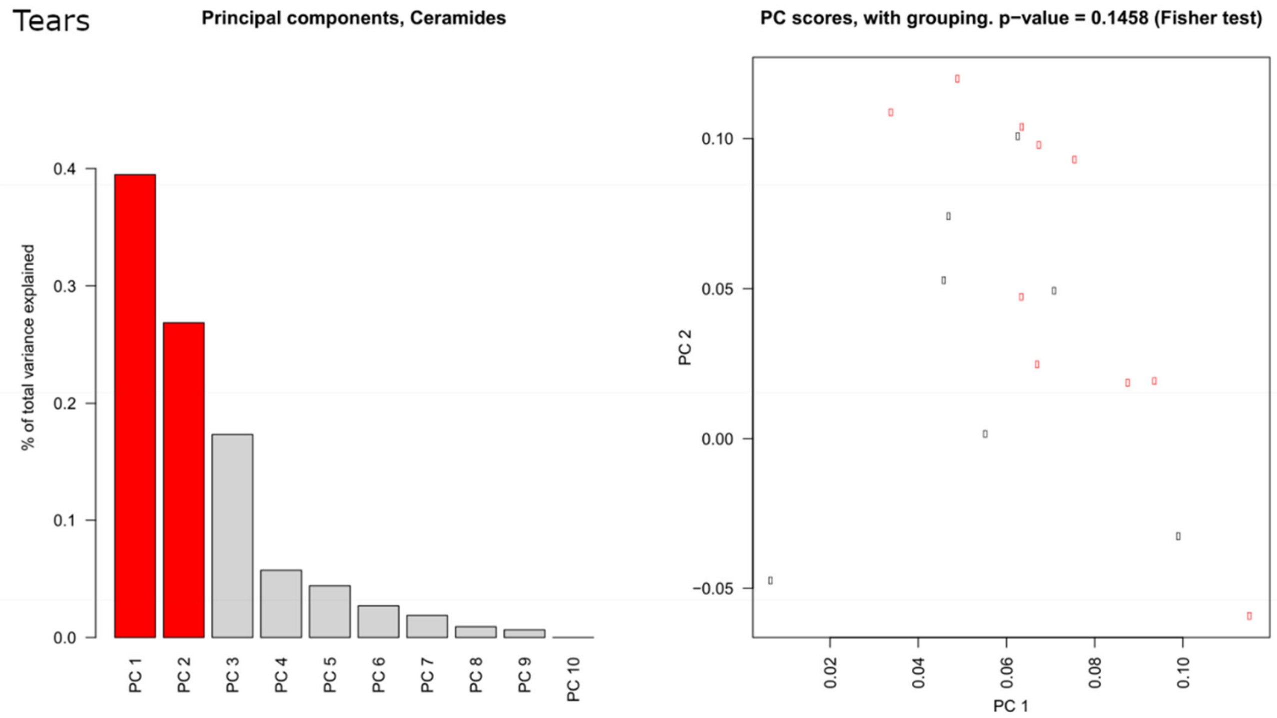

Supplementary Figure S46. Principal component analysis results on ceramide in tears. In the left panel, bar plot of the percentage of the total data variation explained by each estimated principal component. In the right panel, scatterplot of the principal component scores for pSS (•) and control subjects (•). Principal components analysis showed an insignificant difference of p=0.15. PC: principal component.

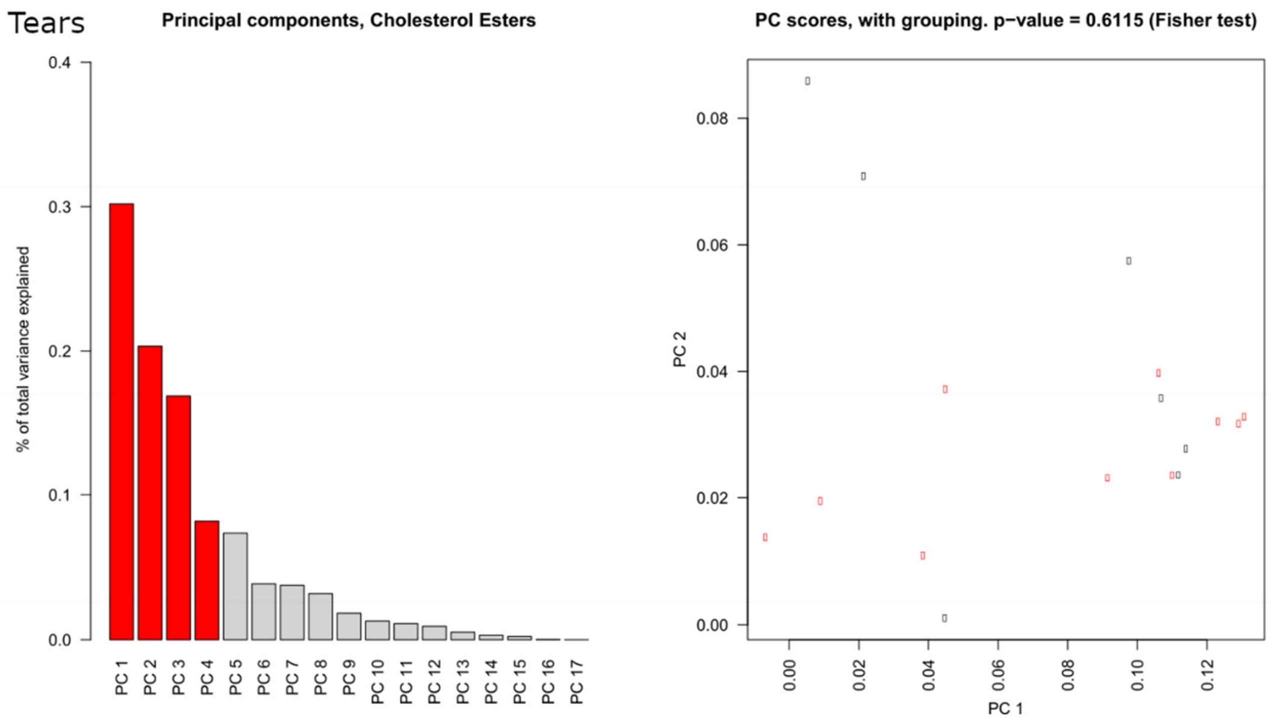

Supplementary Figure S47. Principal component analysis results on cholesterol ester in tears. In the left panel, bar plot of the percentage of the total data variation explained by each estimated principal component. In the right panel, scatterplot of the principal component scores for pSS (•) and control subjects (•). Principal components analysis showed an insignificant difference of p=0.61. PC: principal component.

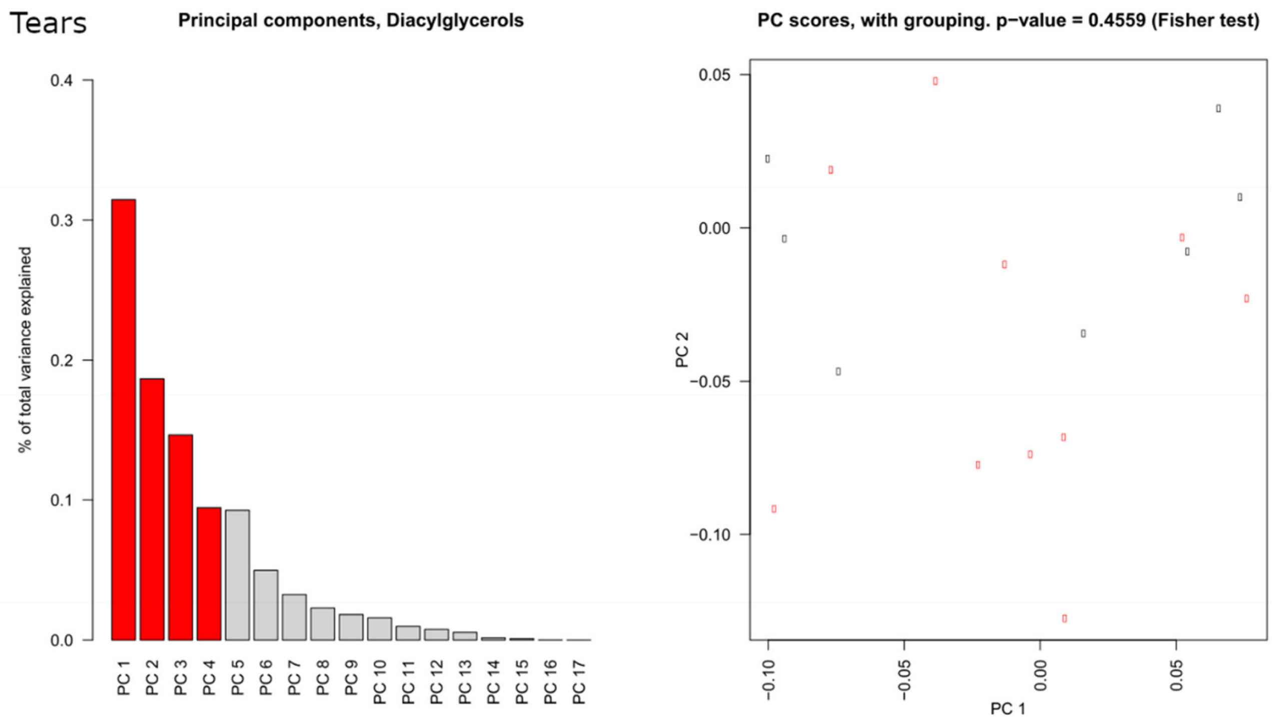

Supplementary Figure S48. Principal component analysis results on diacylglycerol in tears. In the left panel, bar plot of the percentage of the total data variation explained by each estimated principal component. In the right panel, scatterplot of the principal component scores for pSS (•) and control subjects (•). Principal components analysis showed an insignificant difference of p=0.46. PC: principal component.

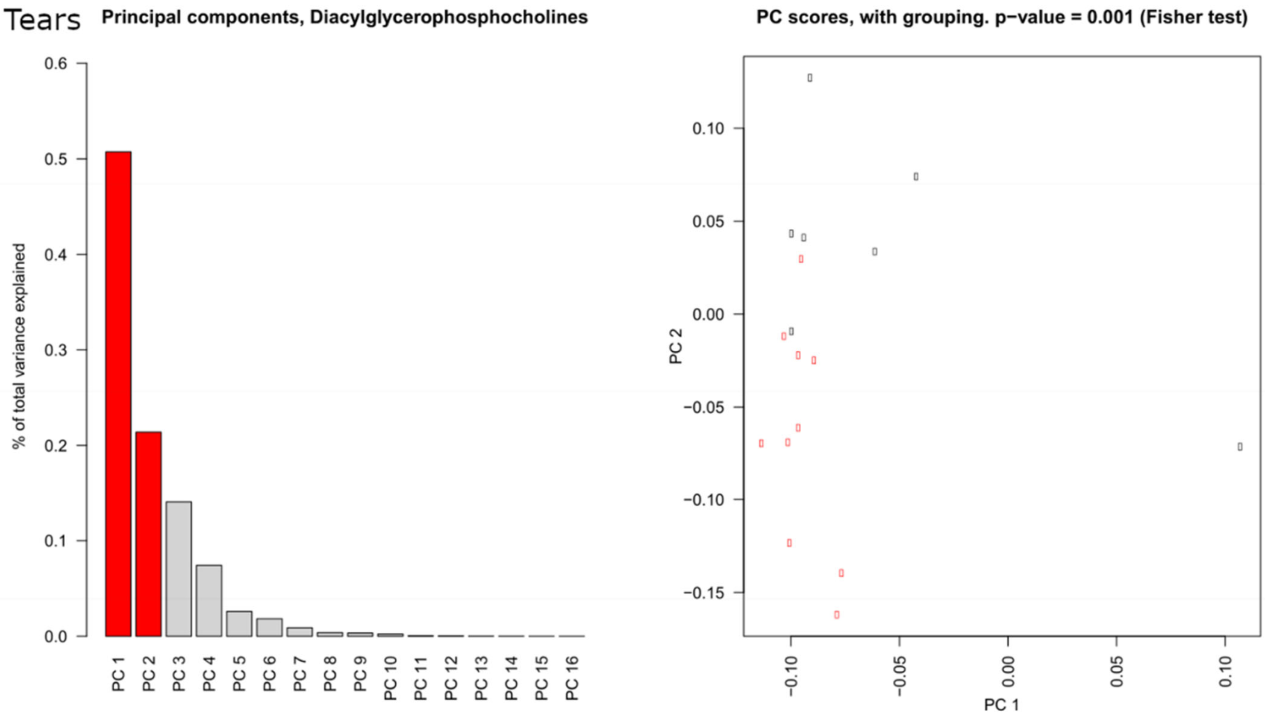

Supplementary Figure S49. Principal component analysis results on diacylglycerophosphocholine in tears. In the left panel, bar plot of the percentage of the total data variation explained by each estimated principal component. In the right panel, scatterplot of the principal component scores for pSS (•) and control subjects (•). Principal components analysis showed a significant difference of p=0.001. PC: principal component.

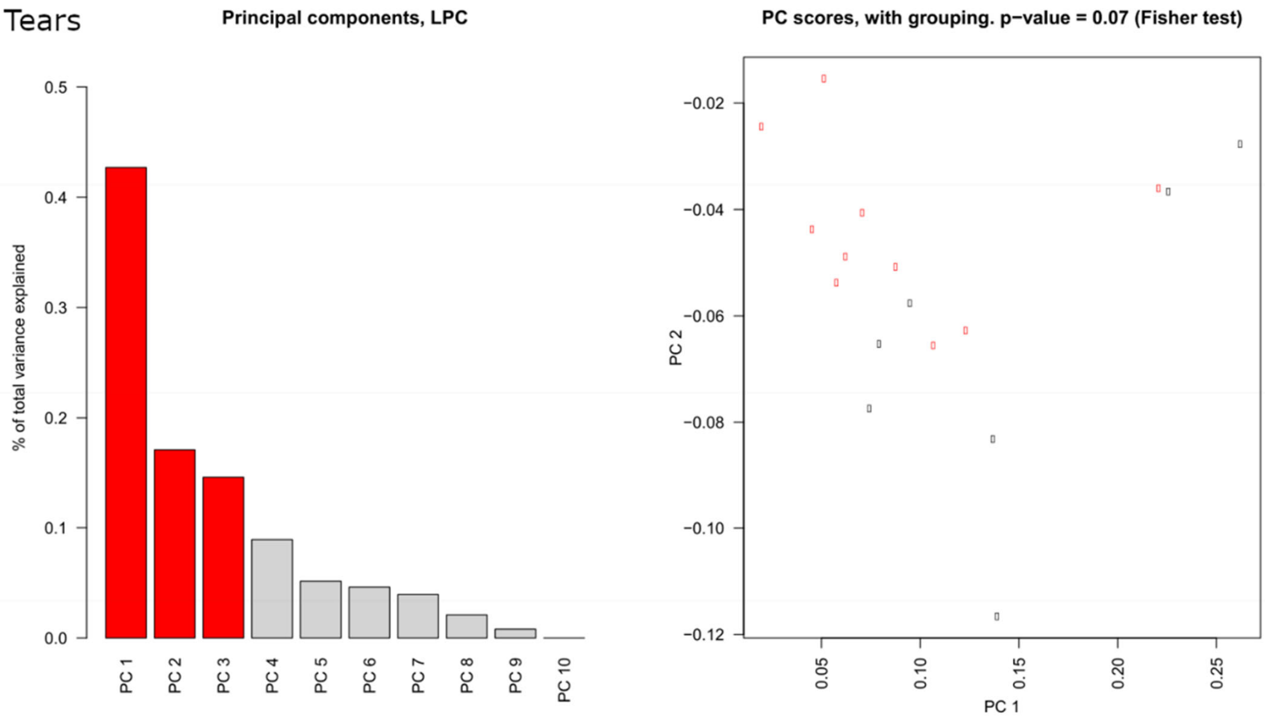

Supplementary Figure S50. Principal component analysis results on lysophosphatidylcholine in tears. In the left panel, bar plot of the percentage of the total data variation explained by each estimated principal component. In the right panel, scatterplot of the principal component scores for pSS (•) and control subjects (•). Principal components analysis showed an insignificant difference of p=0.07. LPC: lysophosphatidylcholine. PC: principal component.

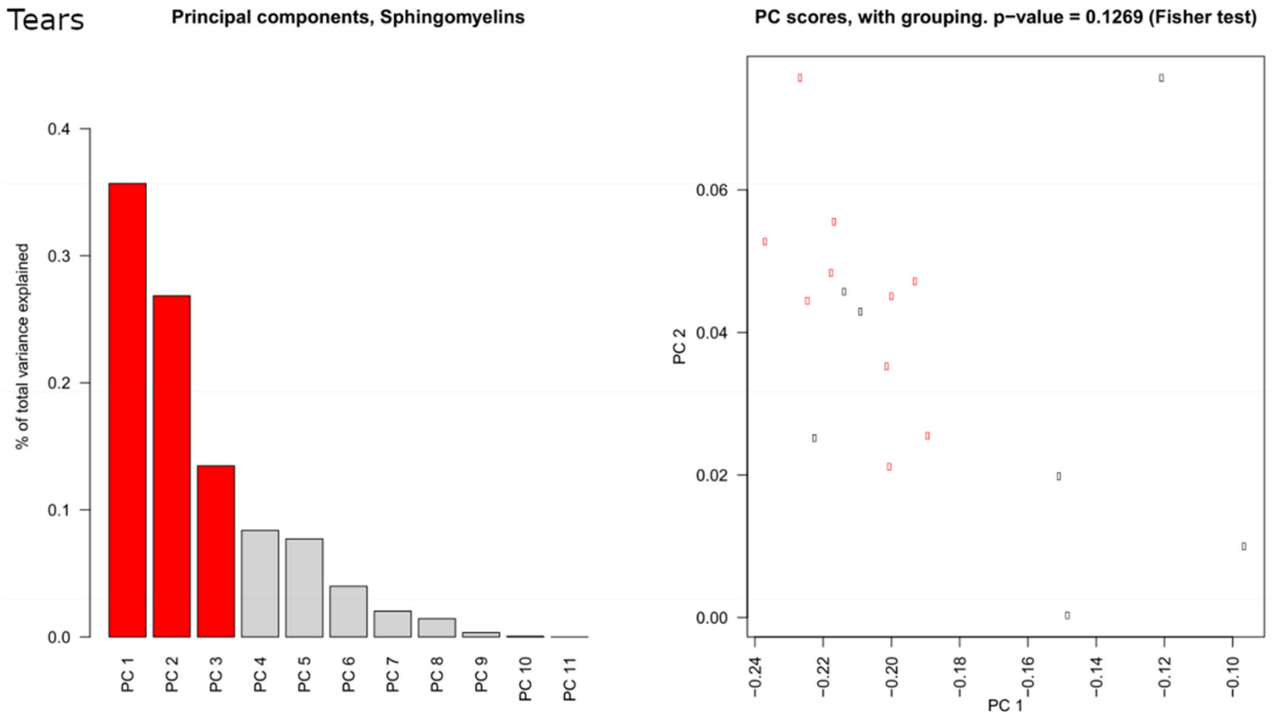

Supplementary Figure S51. Principal component analysis results on sphingomyelin in tears. In the left panel, bar plot of the percentage of the total data variation explained by each estimated principal component. In the right panel, scatterplot of the principal component scores for pSS (•) and control subjects (•). Principal components analysis showed an insignificant difference of p=0.13. PC: principal component.

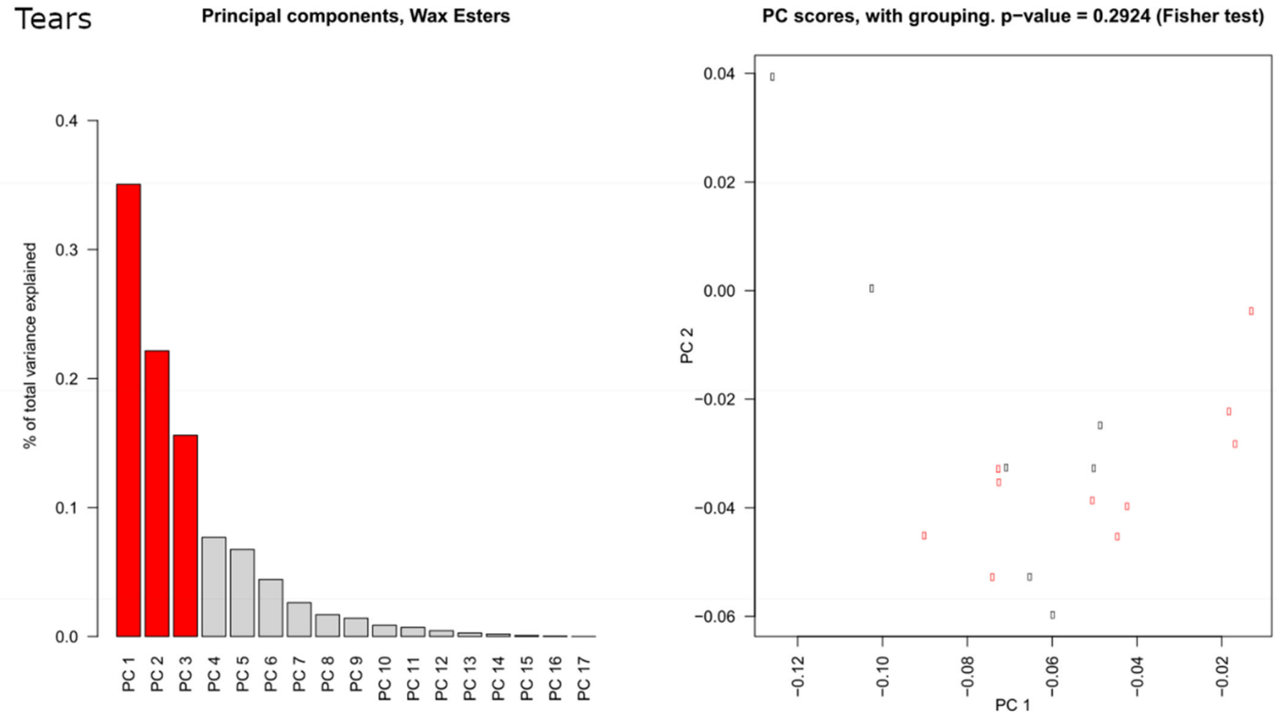

Supplementary Figure S52. Principal component analysis results on wax ester in tears. In the left panel, bar plot of the percentage of the total data variation explained by each estimated principal component. In the right panel, scatterplot of the principal component scores for pSS (•) and control subjects (•). Principal components analysis showed an insignificant difference of p=0.29. PC: principal component.
